# Supplementary material for: COVID-19-related social support service closures and mental well-being in older adults and those affected by dementia: a UK longitudinal survey
Source: BMJ Open. 2021 Jan 17;11(1):e045889. doi: 10.1136/bmjopen-2020-045889 (PMC7813330; doi:10.1136/bmjopen-2020-045889)
Supplement: Author's manuscript [file bmjopen-2020-045889.draft_revisions.pdf]

BMJ Open is committed to open peer review. As part of this commitment we make the peer review history of every article we publish publicly available.

When an article is published we post the peer reviewers' comments and the authors' responses online. We also post the versions of the paper that were used during peer review. These are the versions that the peer review comments apply to.

The versions of the paper that follow are the versions that were submitted during the peer review process. They are not the versions of record or the final published versions. They should not be cited or distributed as the published version of this manuscript.

BMJ Open is an open access journal and the full, final, typeset and author-corrected version of record of the manuscript is available on our site with no access controls, subscription charges or pay-per-view fees (http://bmjopen.bmj.com).

If you have any questions on BMJ Open's open peer review process please email info.bmjopen@bmj.com

BMJ Open

COVID-19-related social support service closures and mental well-being in older adults and those affected by dementia: A UK longitudinal survey

Journal:	*BMJ Open*
Manuscript ID	bmjopen-2020-045889
Article Type:	Original research
Date Submitted by the Author:	27-Oct-2020
Complete List of Authors:	Giebel, Clarissa; University of Liverpool, Institute of Psychology, Health and Society<br>Pulford, Daniel; Lancashire Care NHS Foundation Trust<br>Cooper, Claudia; University College London<br>Lord, Kathryn; University of Bradford<br>Shenton, Justine; Sefton Older People's Forum<br>Cannon, Jacqui; Lewy Body Society<br>Shaw, Lisa; University of Liverpool, Institute of Psychology, Health and Society<br>Tetlow, Hilary; SURF Liverpool<br>Limbert, Stan; NIHR ARC NWC<br>Callaghan, Steve; EQE Health<br>Whittington, Rosie; Me2U Care<br>Rogers, Carol; National Museums Liverpool<br>Komuravelli, Aravind; North West Boroughs Healthcare NHS Foundation Trust<br>Rajagopal, Manoj; Lancashire Care NHS Foundation Trust<br>Eley, Ruth; Liverpool DAA<br>Downs, Murna; University of Bradford, Bradford Dementia Group<br>Reilly, Siobhan; Lancaster University, Division of Health Research<br>Riley, Emma; National Museums Liverpool<br>Ward, Kym; The Brain Charity<br>Gaughan, Anna; TIDE<br>Butchard, Sarah; University of Liverpool, Institute of Psychology, Health and Society<br>Beresford, Jules; University of Bradford<br>Watkins, Caroline; University of Central Lancashire, Faculty of Health and Wellbeing<br>Bennett, Kate; University of Liverpool, Institute of Psychology, Health and Society<br>Gabbay, Mark
Keywords:	Dementia < NEUROLOGY, COVID-19, MENTAL HEALTH, Old age psychiatry < PSYCHIATRY

I, the Submitting Author has the right to grant and does grant on behalf of all authors of the Work (as defined in the below author licence), an exclusive licence and/or a non-exclusive licence for contributions from authors who are: i) UK Crown employees; ii) where BMJ has agreed a CC-BY licence shall apply, and/or iii) in accordance with the terms applicable for US Federal Government officers or employees acting as part of their official duties; on a worldwide, perpetual, irrevocable, royalty-free basis to BMJ Publishing Group Ltd ("BMJ") its licensees and where the relevant Journal is co-owned by BMJ to the co-owners of the Journal, to publish the Work in this journal and any other BMJ products and to exploit all rights, as set out in our licence.

The Submitting Author accepts and understands that any supply made under these terms is made by BMJ to the Submitting Author unless you are acting as an employee on behalf of your employer or a postgraduate student of an affiliated institution which is paying any applicable article publishing charge ("APC") for Open Access articles. Where the Submitting Author wishes to make the Work available on an Open Access basis (and intends to pay the relevant APC), the terms of reuse of such Open Access shall be governed by a Creative Commons licence – details of these licences and which Creative Commons licence will apply to this Work are set out in our licence referred to above.

Other than as permitted in any relevant BMJ Author's Self Archiving Policies, I confirm this Work has not been accepted for publication elsewhere, is not being considered for publication elsewhere and does not duplicate material already published. I confirm all authors consent to publication of this Work and authorise the granting of this licence.

For peer review only

COVID-19-related social support service closures and mental well-being in older adults and those affected by dementia: A UK longitudinal survey

Clarissa Giebel[1,2,*], Daniel Pulford[3], Claudia Cooper[4], Kathryn Lord[5], Justine Shenton[6], Jacqueline Cannon[7], Lisa Shaw[8], Hilary Tetlow[9], Stan Limbert[2], Steve Callaghan[10], Rosie Whittington[11], Carol Rogers[12], Aravind Komuravelli[13], Manoj Rajagopal[3], Ruth Eley[14], Murna Downs[5], Siobhan Reilly[5], Emma Riley[12], Kym Ward[15], Anna Gaughan[16], Sarah Butchard[17], Jules Beresford[5], Caroline Watkins[2,18], Kate Bennett[19], Mark Gabbay[1,2]

1 Department of Primary Care & Mental Health, University of Liverpool, UK
2 NIHR ARC NWC, Liverpool, UK
3 Lancashire and South Cumbria NHS Trust, UK
4 Division of Psychiatry, UCL, UK
5 Centre for Applied Dementia Studies, University of Bradford, UK
6 Sefton Older People's Forum, Sefton, UK
7 Lewy Body Society, Wigan, UK
8 Department of Modern Languages and Cultures, University of Liverpool, UK
9 SURF Liverpool, UK
10 EQE Health, Liverpool, UK
11 Me2U Care, Liverpool, UK
12 National Museums Liverpool, UK
13 North West Boroughs NHS Trust, Warrington, UK
14 Liverpool Dementia Action Alliance, UK
15 The Brain Charity, Liverpool, UK
16 TIDE, Liverpool, UK
17 Mersey Care NHS Trust, Liverpool, UK
18 Department of Health Research, UCLAN, UK
19 School of Psychology, University of Liverpool, UK

**Correspondence should be addressed to:* Clarissa Giebel, Waterhouse Building, University of Liverpool, Liverpool, L69 3GL. Email: Clarissa.giebel@liverpool.ac.uk

For peer review only

For peer review only - http://bmjopen.bmj.com/site/about/guidelines.xhtml

Abstract

Background: The COVID-19 pandemic has had a major impact on delivery of social support services. This might be expected to particularly affect older adults and people living with dementia (PLWD), and to reduce their wellbeing.

Aims: To explore how social support service use by older adults, carers, and PLWD, and their mental wellbeing changed over the first three months since the pandemic outbreak.

Methods: Unpaid dementia carers, PLWD, and older adults took part in a longitudinal online or telephone survey collected in April-May 2020, and at two subsequent time points 6 and 12 weeks after baseline. Participants were asked about their social support service usage in a typical week prior to the pandemic (at baseline), and in the past week at each of the three time points. They also completed measures of depression, anxiety and mental well-being.

Results: 377 participants had complete data at all three time points. Social support service usage dropped shortly after lockdown measures were imposed at timepoint 1 (T1), to then increase again by T3. The access to paid care was least affected by COVID-19. Cases of anxiety dropped significantly across the study period, whilst cases of depression rose. Well-being increased significantly for older adults and PLWD from T1 to T3.

Conclusions: Access to social support services has been significantly affected by the pandemic, which is starting to recover slowly. With mental well-being differently affected across subgroups, support needs to be put in place to maintain better well-being across those vulnerable groups during the ongoing pandemic.

For peer review only

Strengths and limitations of this study

- Data on social support service usage and mental health were collected at three time points in the early stages of the pandemic.
- 63% of participants completed all three survey time points.
- The survey provides a unique insight into how social care and mental health have been affected in dementia and ageing during COVID-19.
- The study was mostly completed by people from a White ethnic background, and lacks ethnic minority representation despite actively approaching community groups.

For peer review only

For peer review only - http://bmjopen.bmj.com/site/about/guidelines.xhtml

Introduction

In the UK, 11.9 million people are aged 65 and over [1], with over 850,000 living with dementia [2]. Social support services, including day care centres, support groups, paid home carers, and community activities, such as singing or arts groups, are important for maintaining a good quality of life for older people and people living with dementia (PLWD) [3-4]. In view of an ageing population and increasing numbers of PLWD, easily accessible services are crucial to support people socially, as well as with their care needs.

The COVID-19 pandemic has affected these social support services significantly. Social isolation as a result of social distancing, lockdowns, and shielding is a huge concern for older people across the globe since the beginning of the pandemic [5-6], with similar issues highlighted early for PLWD [7]. However, there is still a dearth of evidence on the mental well-being and access to care for those in need.

Specifically, in the UK, a nationwide three-month lockdown was imposed on the 23rd of March. Older people were over-represented in the group who were clinically extremely vulnerable, that is at greatest risk of severe illness from COVID-19, who were asked to shield by the government until early August. All adults aged 70+ were classified as being at least moderate risk of severe illness from COVID-19 [8]. During the most restrictive, earlier period of lockdown, people were advised to only go outside once a day for essential food shopping, pharmacy visits, or to exercise. Non-essential shops were closed, and only started reopening in July. With additional social distancing for the general population, and use of personal protective equipment (PPE) for the health and social care taskforce in place, these measures significantly impact the social support services that PLWD, carers, and older adults could receive. Recent qualitative evidence has highlighted how PLWD and unpaid carers have faced a sudden crisis in terms of accessing social support services since the pandemic [9], and have faced difficult decisions whether to continue or discontinue paid carers entering the home of the PLWD, for fear of potential virus transmission [10]. Whilst these qualitative accounts provide rich information on the experiences of having accessed (or failed to access) social support services during the pandemic, there appears to be no empirical evidence to date quantifying those experiences and linking these with mental well-being.

The aim of this study was to explore the impacts of COVID-19 on social support service closures and longitudinal changes in the mental illness and well-being of older adults, PLWD, and unpaid carers.

Methods

Participants and recruitment

We recruited UK residents who were aged 18+. PLWD were eligible to take part if they had a diagnosis of dementia. Unpaid carers were eligible to take part if they were or had been caring for a relative or friend with dementia. Older adults were eligible to take part if they were aged 65 years or older.

Participants were recruited via different social support services third sector organisations, such as peer support group organisations, carer networks, cultural dementia training programme organisations, and national dementia subtype specific organisations, and

For peer review only - http://bmjopen.bmj.com/site/about/guidelines.xhtml

by contacting people on their email circulation lists, via newsletters and social media accounts. We also directly contacted people who were accessing regular services, such as support groups or older people fora, via telephone. This ensured that people without internet access were able to participate in this research. We also utilised *Join Dementia Research*, a UK-wide national online register of PLWD, carers, older adults, and health volunteers who are interested in taking part in dementia and ageing research.

Ethical approval was obtained from the University of Liverpool prior to study begin [Ref: 7626].

Data collection

The study was completed at three time points (T1, T2, T3), 5 and 6 weeks apart, respectively. Participants could complete the survey either online or over the phone with a research team member who entered their details into the online survey on their behalf. Participants from T1 (baseline) were followed-up with the same mental well-being questionnaires at T2 and T3 and were followed-up either by telephone or email, depending on how they completed T1 survey. T1 ran from 17th April to 15th May (+/- 3 days). T2 ran from 29th May to 26th June (+/- 3 days). T3 ran from 10th July to 7th August (+/- 3 days).

Variables and tools

At T1, participants were asked about their background characteristics (including age, gender, ethnicity, postcode, living situation, type of dementia (if applicable), and employment). Postcode data were collected to generate an Index of Multiple Deprivation (IMD) quintile. IMD provides a measure of neighbourhood deprivation, taking into account income, education, crime, and health, amongst others. Quintile 1 indicates least deprived neighbourhoods, with quintile 5 indicating the most deprived neighbourhoods.

Service usage was measured by asking about pre-pandemic and current receipt of different social support services (including paid carers, support groups, befrienders, day care centres, respite, meal deliveries, transport, social activities, clinical mental health support, and clinical physical support) and equipment, such as hand rails or shower seats, as well as the weekly total hours of social support services. Pre-pandemic service usage was defined as use of social support services in a typical week before the pandemic.

Participants were also asked to complete the Personalised Health Questionnaire 9 (PHQ-9) [11] for depression, the Generalised Anxiety Disorder 7 (GAD-7) [12] for anxiety, and the Short Warwick-Edinburgh Mental Well-Being Scale [13] (SWEMWBS) for quality of life. Higher scores on the PHQ-9, GAD-7, and the SWEMWBS indicated higher levels of depression, anxiety, and quality of life, respectively. We categorised participants who scored of 10 or more on the PHQ-9 as 'depressed' and on the GAD-7 as 'anxious' [14]. At T2 and T3, participants were asked again about their current levels of social support service receipt, weekly hours of support, equipment, as well as the PHQ-9, GAD-7, and SWEMWBS.

Data analysis

Data were analysed using SPSS 25, and the significance level was set at $p<0.05$. Participant demographic characteristics and social support service usage and mental well-being variables were analysed using frequency analysis. Chi-square tests were used to assess variations in the proportions of participants who were categorised as 'depressed' or 'anxious'. Repeated measures ANOVAs with Greenhouse Geisser posthoc correction were used to analyse differences between T1, T2, and T3 in GAD-7 total, PHQ-9 total, and SWEMWBS total scores.

For peer review only - http://bmjopen.bmj.com/site/about/guidelines.xhtml

For this analysis, only participants with complete GAD-7, PHQ-9, and SWEMWBS were included (n=377).

Public involvement

Unpaid carers and a PLWD were involved as equal team members in all aspects of the study – from conceptualisation and design through to analysis and dissemination.

Results

Survey completion

Figure 1 outlines the participant flow and completion rates in further detail. 569 participants completed the survey at T1 (61 PLWD; 219 current carers; 66 former carers; 223 older adults). 420 participants completed the survey at T2 (38 PLWD; 168 current carers; 45 former carers; 169 older adults). 377 participants completed all three waves of the survey (37 PLWD; 147 current carers; 42 former carers; 148 older adults).

Participant characteristics

Table 1 shows the demographic characteristics of those who completed T1 and those that completed all three survey time points, by subgroup. For those who completed all three time points, carers and older adults were mostly female (59-82%), whilst the majority of PLWD were male (62%). The majority of participants were from a White ethnic background (95-99%) and lived with someone else (61-88%), with current carers having the highest proportion of living with someone else. The majority of participants across all four subgroups lived in less deprived neighbourhoods (Quintiles 1 and 2) (52-61%). Thirty-seven PLWD took part in all three time points; the most common diagnostic subtype was Alzheimer's disease.

[Figure 1 and Table 1]

Social support service and activities usage

Participants had accessed a range of social support services pre-pandemic, including day care centres, support groups, meal deliveries, respite, and paid carers. Figure 2 (A) shows the proportion of participants of the total sample (n=377) who reported accessing paid carers, support groups, day care, befrienders, and social activities prior to the pandemic, and at T1, T2, and T3. These were the most commonly used types of social support services prior to the pandemic. Social support services usage had dropped since the pandemic outbreak. Pre-pandemic, 27% of participants accessed social activities in the community, which dropped to 6% at T1, T2, and T3. Paid care saw the smallest change – with 17% having accessed paid carers pre-pandemic, dropping to 12% at T1 and increasing slightly again to 15% at T3. Day care saw the largest drop, with only 1-2 % receiving day care since the outbreak, compared to 15% previously.

Figure 2 (B) shows the proportion of participants by group who have received any form of social support services pre-pandemic and at all three survey time points. Pre-pandemic, 90% of current carers had received social support of any form, with between 45% and 50% of former carers and older adults having received some support. This decreased at T1 for all subgroups to between 20% (older adults) to 55% (current carers) receiving some type of support. Through T2 and T3, an upward trend emerged with more participants gaining access to some services again, with levels for PLWD and former carers being higher at T3 than at pre-pandemic levels.

For peer review only - http://bmjopen.bmj.com/site/about/guidelines.xhtml

[Figure 2]

Mental well-being

Figure 3 shows the proportion of participants across the total sample (n=377) who were categorised as anxious and depressed across all three time points. For anxiety, we noted a downward trend in number of cases from T1 (16.5%) to T3 (14.1%). The proportion of participants with anxiety was significantly lower at T2 (x^2=186.399, p<0.001) and T3 (x^2=136.562, p<0.001) compared to T1. For depression, we noted an upward trend in cases from T1 (14.4%) to T3 (17.5%). The proportion of participants with depression was significantly higher at T2 (x^2= 176.248, p<0.001) and T3 (x^2=158.031, p<0.001) compared to T1.

Figure 4 shows the median of the GAD-7, PHQ-9, and SWEMWBS total scores by group over time for those who completed all three survey time points. Based on the median scores, levels of anxiety and depression appear to decrease from T1 to T3, whilst quality of life increases from T1 to T3.

For anxiety, repeated-measures ANOVAs with Greenhouse-Geisser posthoc correction showed that GAD-7 total scores did not vary significantly from T1 to T3 for PLWD [F(1.856, 64.962)= 1.429, p= 0.247]or among current carers [F(1.898, 277.063)= 1.938, p=0.148], former carers[F(1.801, 68.419)=.139, p=.139], or older adults [F(1.924, 286.727)= 2.688, p=.0072], based on those participants who completed in all three survey time points.

For depression, repeated-measures ANOVAs showed that PHQ-9 total scores did not significantly vary from T1 to T3 for PLWD [F(1.896, 66.370)= 1.461, p=0.240], current carers [F(1.900, 277.453)= .639, p=0.521], former carers [F(1.677, 68.419)= .024, p=0.960], or older adults [F(1.889, 281.414)= .857, p=0.420].

For well-being, repeated-measures ANOVAs showed that SWEMWBS total scores significantly increased from T1 to T3 for PLWD [F(1.726, 60.423)= 5.412, p<0.05 [Mean(SD) T1-T3= 22.1(6.4); 24.3(5.6); 24.4(5.6)]] and older adults [F(1.804, 268.807)= 3.632, p<0.05 [Mean(SD) T1-T3= 27.5(5.1); 28.0(5.1); 28.3(4.9)]]. However there were no significant changes among current [F(1.982,289.325)= 2.185, p=0.115] or former carers [F(1.728, 63.725)= .268, p=0.733].

[Figure 3 and 4]

Discussion

This is one of the first studies to show that social support service usage in dementia and ageing reduced significantly compared to pre-pandemic levels, whilst slowly rising in the months post-nationwide lockdown. In addition, we also show that cases of anxiety reduced whilst cases of depression increased in the months since lockdown, with quality of life significantly increasing for PLWD and older adults only.

Social support service usage for PLWD, unpaid carers, and older adults has seen a significant decrease since the COVID-19 pandemic, leaving many people suddenly without vital support – ranging from day care centres to respite to support groups. In the months following the nationwide lockdown, usage has gradually increased again but varied among providers and type of support. With public health restrictions still remaining in place however during that period, including social distancing, shielding and thus inability to meet members of different households, such support is most likely to be implemented via digital technologies. Considering that in our sample 94% of participants completed the survey online rather than the telephone option, nearly all participants had access to the internet. However, many older adults and PLWD are less likely to be digitally literate [15], making it difficult for all people to access services equally. This has already been an issue pre-COVID-19 [16], suggesting that the pandemic has further exacerbated potential inequalities in access and thus further isolated people who would benefit from social support the most.

For peer review only - http://bmjopen.bmj.com/site/about/guidelines.xhtml

One type of support which has been affected the least by the pandemic has been paid home care. Receiving paid home care enables PLWD and older adults to stay at home independently for longer – as people wish to avoid entering a care home and stay in their familiar environment [17]. Whilst there was a reduction in paid home care usage compared to pre-pandemic levels, overall paid home carers were utilised the most. A qualitative exploration into decision-making for whether or not to continue paid home care during the pandemic has shown that many unpaid carers were afraid of having paid carers enter the home (often with inadequate PPE) for risk of potential virus transmission [10]. Other unpaid carers however felt unable to cope without the support, or indeed accepted the potential risks, and continued paid home care. There is also a notable difference between social care provision (which is paid home care) and third sector care provision (which involves support groups and social activities for example). The third sector relies on volunteers providing services, and has also suffered during the pandemic, whereas the social care sector is financially supported by the government. Therefore, the ability to receive home care might not have been affected to the same extent as accessing support groups for instance. Another potential reason for variations in usage between activity types is that home care involves someone from the outside entering someone's home. In contrast, day care centres, respite care, and social activities involve older adults and PLWD going outside to larger social gatherings. Due to public health restrictions, these have been temporarily rendered largely, if not completely, impossible to take place in their original face-to-face formats. As numbers of infections rise again, these restrictions are being strengthened and re-imposed with large fines possible for those transgressing them.

Anxiety, depression, and well-being changed over the course of the study period. Over 12 weeks, cases of anxiety across the total sample dropped, whilst cases of depression increased significantly. However, when exploring levels of anxiety and depression within groups, no significant changes were noted, which is likely to have been due to small and varied sample sizes for each subgroup. This may be because people who were very depressed or anxious might not have continued the follow-up surveys. It is also possible though that participants felt more connected over time, particularly considering again that the majority of participants completed the survey online and thus were able to participate in remote services, where these existed. Recent evidence from Spain showed how older adults were less likely to suffer from psychological distress as a result of the pandemic than people aged below 60 [18]. Nevertheless, overall the pandemic is having a heightened impact on the mental health of the general population [19-20]. Engaging in social activities can be one avenue to help maintain good mental health [21]. Considering that reductions in social engagement both before and after a dementia diagnosis are common [22], enabling continued engagement throughout the pandemic is important to support PLWD, carers, and older adults adequately. This is corroborated by evidence from the baseline survey showing that reductions in social support usage were linked to mental well-being [23]. It is possible that for this study, merging subgroups of older adults, PLWD, and carers resulted in no significant associations, as each group was differently affected, as indicated by looking at changes of mental well-being for each group across the 12 weeks.

There were some limitations to our study. Whilst benefitting from a large sample size and good retention rate over a relatively short time period of 12 weeks, the majority of participants had internet access and were thus also able to join in remote social support. Although we actively approached older adults, PLWD, and carers via phone through recruiting organisations, only some people took part over the phone. It is likely however that those people without internet access have been even more isolated through the pandemic, with potentially severe mental health needs, which we have only captured a snapshot of. This also links to the fact that there are likely to be longer-term effects on mental well-being, with our survey only providing a snap shot of the first few months since the start of the pandemic. Equally, our survey did not include pre-pandemic levels of mental well-being (anxiety,

For peer review only - http://bmjopen.bmj.com/site/about/guidelines.xhtml

depression, and quality of life), which would have provided additional insight into changes in mental well-being. However, due to the unforeseen circumstance of the pandemic, it was not feasible to collect these data. We only enquired about weekly hours of total social support usage, and not for each specific type of activity. Some participants might have accessed, for example, paid home care, but only for two hours as opposed to others who might have received 40 hours a week. We are thus unable to state in detail how the pandemic has affected the level of each different type of support, but instead we provide a more general overview of activities and general service usage variations since the nationwide lockdown, which to existing knowledge has not been captured elsewhere.

Conclusions

The pandemic is having a sudden and severe long-term impact on social support service usage for older adults and people affected by dementia, which sees somewhat of a limited increase in usage over the first few months since nationwide lockdown. Whilst it appears that some services have started providing remote support, not everyone will be able to access these, leaving many people without much needed support. Future research needs to assess how older adults and people affected by dementia are accessing social support services in the time of COVID-19, with clearer support for people to access any format of services – either face-to-face or remotely. Considering that the pandemic is going to continue for the foreseeable future, the mental health of older adults and those affected by dementia needs to be closely monitored, particularly when more stringent public health measures are put in place again.

For peer review only

Conflicts of interest
None.

Acknowledgements
This study would not have been possible without our participants, thank you very much. We also wish to thank Join Dementia Research for helping us recruit to our study.

Funding
This research is supported by a grant awarded to the authors by the University of Liverpool COVID-19 Strategic Research Fund in 2020. This is also independent research funded by the National Institute for Health Research Applied Research Collaboration North West Coast (ARC NWC). The views expressed in this publication are those of the author(s) and not necessarily those of the National Institute for Health Research or the Department of Health and Social Care. The University of Bradford QR Research Fund also supported part of this study. There are no grant numbers.

For peer review only

Figure Captions

Figure 1. Flow of participation in longitudinal survey

NOTE. The top boxes indicate how many people completed each survey time point. After having removed duplicates, missing cases (where participants had not completed the PHQ-9, GAD-7, and the SWEMWBS or had missing ID codes at T2 and T3), and those that had completed T2 and T3 but had not completed T1, 377 cases remained in total. Grey boxes indicate the breakdown by subgroup.

[1] Follow-up completion by subgroup by percentage at T2 and T3 compared to T1: PLWD 69%(T2), 67%(T3); Current Carers 86%(T2), 75%(T3); Former carers 64%(T2), 60%(T3); Older adults 79%(T2), 69%(T3).

Figure 2. Social support service usage pre-pandemic and at 3 survey time points

Note. (A) Service usage for the total sample (N=377) in proportion of participants at four different time points for some of the most frequently used support services. (B) Proportion of participants within each group at four different timepoints (pre-pandemic, T1, T2, T3) having received any form of social support.

Figure 3. Proportion of the total sample who scored above the cut offs for anxiety and depression at three time points

NOTE. T = Time point

The graph shows the proportion of participants from the total sample who completed all three surveys (n=377) and scored above the cut off on the GAD-7 and PHQ-9 for anxiety and depression, respectively.

Figure 4. Variations in anxiety, depression, and quality of life total scores at 3 time points

NOTE. Figures show the median total score at each time point (T1, T2, T3) for each subgroup – for anxiety (GAD-7), depression (PHQ-9), and quality of life (SWEMWBS).

For peer review only - http://bmjopen.bmj.com/site/about/guidelines.xhtml

References

[1] Office for National Statistics (ONS). *Living longer: is age 70 the new age 65?* London, England 2019: ONS.

[2] Alzheimer's Society. *Dementia UK: An Update*. London, England: Alzheimer's Society 2014.

[3] Skingley A, Martin A, Clift S. The contribution of community singing groups to the well-being of older people: participant perspectives from the United Kingdom. Journal of Applied Gerontology 2016;35(12):1302-1324.

[4] Tretteteig S, Vatne S, Mork Rokstad AM. The influence of day care centres designed for people with dementia on family caregivers – a qualitative study. BMC Geriatrics 2017;17(5).

[5] Armitage R, Nellums LB. COVID-19 and the consequences of isolating elderly. LANCET Public Health 2020; 5(5):doi: 10.1016/S2468-2667(20)30061-X

[6] Berg-Weger M, Morley JE. Loneliness and social isolation in older adults during the COVID-19 pandemic: implications for gerontological social work. Journal of Nutrition, Health & Ageing 2020;24(5):456-458.

[7] Brown EE, Kumr S, Rajji TK, et al. Anticipating and mitigating the impact of the COVID-19 pandemic on Alzheimer's disease and related dementias. American Journal of Geriatric Psychiatry 2020;28(7):712-721.

[8] Public Health England (PHE). *Guidance on shielding and protecting people who are clinically extremely vulnerable from COVID-19.* London, England 2020: PHE. Last accessed 22nd September 2020: https://www.gov.uk/government/publications/guidance-on-shielding-and-protecting-extremely-vulnerable-persons-from-covid-19/guidance-on-shielding-and-protecting-extremely-vulnerable-persons-from-covid-19#definition

[9] Giebel C, Cannon J, Hanna K, et al. Impact of Covid-19 related social support service closures on people with dementia and unpaid carers: A qualitative study. Aging & Mental Health 2020a; doi: 10.1080/13607863.2020.1822292

[10] Giebel C, Hanna K, Cannon J, et al. Decision-making for receiving paid home care for dementia in the time of COVID-19: A qualitative study. BMC Geriatrics 2020b; doi: 10.1186/s12877-020-01719-0

[11] Kroenke K, Spitzer RL, Williams JB. The PHQ-9: validity of a brief depression severity measure. Journal of General Internal Medicine 2001;16(9):606-613.

[12] Spitzer RL, Kroenke K, Williams JB, Loewe B. A brief measure for assessing generalized anxiety disorder: the GAD-7. Archives of Internal Medicine 2006;166(10):1092-1097.

[13] Haver A, Akerjordet K, Caputi P, et al. Measuring mental well-being: A validation of the Short Warwick-Edinburgh Mental Well-Being Scale in Norwegian and Swedish. Scandinavian Journal of Public Health 2015;43(7):721-727.

[14] Smith, B.W., Epstein, E.E., Oritz, J.A., Christopher, P.K., & Tooley, E.M. (2013). The Foundations of Resilience: What are the critical resources for bouncing back from stress? In Prince-Embury, S. & Saklofske, D.H. (Eds.), *Resilience in children,*

For peer review only - http://bmjopen.bmj.com/site/about/guidelines.xhtml

adolescents, and adults: Translating research into practice, The Springer series on human exceptionality (pp. 167-187). New York, NY: Springer.

[15] Hill R, Betts LR, Gardner SE. Older adults' experiences and perceptions of digital technology: (Dis)empowerment, wellbeing, and inclusion. Computers in Human Behavior 2015;48: 415-423.

[16] Stephan A, Bieber A, Hopper L, et al. Barriers and facilitators to the access to and use of formal dementia care: findings of a focus group study with people with dementia, informal carers and health and social care professionals in eight European countries. BMC Health Services Research 2018; 131: doi: 10.1186/s12877-018-0816-1

[17] Rapaport P, Burton A, Leverton M, et al. "I just keep thinking that I don't want to rely on people." A qualitative study of how people living with dementia achieve and maintain independence at home: stakeholder perspectives BMC Geriatrics 2020; doi: 10.1186/s12877-019-1406-6

[18] Garcia-Portilla P, de la Fuente Tomas L, Bobes-Bascaran T, et al. Are older adults also at higher psychological risk from COVID-19? Aging & Mental Health 2020; doi: 10.1080/13607863.2020.1805723

[19] Fancourt D, Steptoe A, Bu F. Trajectories of depression and anxiety during enforced isolation due to COVID-10: longitudinal analyses of 59,318 adults in the UK with and without diagnosed mental illness. medRxiv preprint 2020; doi: 10.1101/2020.06.03.20120923

[20] Iob E, Steptoe A, Fancourt D. Abuse, self-harm and suicidal ideation in the UK during the COVID-19 pandemic. British Journal of Psychiatry 2020; doi: 10.1192/bjp.2020.130

[21] Holtfreter K, Reisig MD, Turanovic JJ. Depression and infrequent participation in social activities among older adults: the moderating role of high-quality familial ties. Aging & Mental Health 2017;21(4): 10.1080/13607863.2015.1099036

[22] Hackett RA, Steptoe A, Cadar D, Fancourt D. Social engagement before and after dementia diagnosis in the English Longitudinal Study of Ageing. PLOS One 2019; doi: 10.1371/journal.pone.0220195

[23] Giebel C, Lord K, Cooper C, et al. A UK survey of COVID-19 related social support service closures and the effects on older people, people with dementia, and carers. International Journal of Geriatric Psychiatry 2020c; doi: 10.1002/gps.5434

For peer review only - http://bmjopen.bmj.com/site/about/guidelines.xhtml

Table 1. Participant characteristics of those completing T1 survey and those completing all three survey time points

	T1 (n=569)				T1, 2 and 3 (n=377)			
	PLWD (n=61)	Current carers (n=219)	Former carers (n=66)	Older adults (n=223)	PLWD (n=37)	Current carers (n=149)	Former carers (n=39)	Older adults (n=152)
**N(%)**								
**Gender**								
**Female**	27 (44.3)	168 (77.1)	55 (83.3)	137 (61.7)	14 (37.8)	118 (79.7)	32 (82.1)	90 (59.2)
**Male**	34 (55.7)	50 (22.9)	11 (16.7)	85 (38.3)	23 (62.2)	30 (20.3)	7 (17.9)	62 (40.8)
**Ethnicity**								
**White**	58 (96.7)	211 (96.3)	65 (98.5)	216 (98.2)	35 (94.6)	143 (96.0)	38 (97.4)	148 (98.7)
**Other**	2 (3.4)	8 (3.7)	1 (1.5)	4 (1.9)	2 (5.4)	6 (4.0)	1 (2.6)	2 (1.3)
**Living situation**								
**Alone**	13 (21.3)	33 (15.1)	17 (26.2)	79 (35.6)	8 (21.6)	18 (12.2)	11 (28.9)	59 (39.1)
**With someone**	48 (78.7)	185 (84.9)	48 (73.8)	143 (64.4)	29 (78.4)	130 (87.8)	27 (71.1)	92 (60.9)
**IMD Quintile**								
**1**	12 (23.1)	54 (32.1)	10 (19.2)	61 (33.5)	5 (16.1)	35 (31.0)	6 (19.4)	39 (31.7)
**2**	16 (30.8)	50 (29.8)	20 (38.5)	44 (24.2)	11 (35.5)	34 (30.1)	11 (35.5)	29 (23.6)
**3**	10 (19.2)	32 (19.0)	14 (26.9)	37 (20.3)	6 (19.4)	20 (17.7)	9 (29.0)	26 (21.1)
**4**	10 (19.2)	14 (8.3)	5 (9.6)	26 (14.3)	5 (16.1)	11 (9.7)	4 (12.9)	18 (14.6)
**5**	4 (7.7)	18 (10.7)	3 (5.8)	14 (7.7)	4 (12.9)	13 (11.5)	1 (3.2)	11 (8.9)
**Type of dementia**								
**Alzheimer's**	20 (32.8)	100 (46.5)	6 (23.1)		14 (37.8)	75 (50.7)	2 (20.0)	
**Mixed**	13 (21.3)	49 (22.8)	7 (26.9)		6 (16.2)	34 (23.0)	4 (40.0)	
**Vascular**	11 (18.0)	27 (12.6)	4 (15.4)		8 (21.6)	18 (12.2)	3 (30.0)	
**Other**	17 (27.9)	39 (18.1)	9 (34.5)		9 (24.3)	21 (14.1)	1 (10.0)	
**Mean (SD), [Range]**								
**Age**	70 (+/-10), [45-88]	61 (+/-13), [23-89]	64 (+/-14), [22-95]	72 (+/-6), [65-90]	72 (+/-10), [50-88]	62 (+/-13), [23-89]	65 (+/-13), [22-95]	73 (+/-6), [65-90]
**Years of education**	15 (+/-4), [4-25]	16 (+/-4), [6-28]	17 (+/-4), [10-29]	17 (+/-4), [7-25]	13 (+/-4), [4-20]	16 (+/-4), [6-28]	16 (+/-4), [10-29]	16 (+/-4), [7-24]

NOTE. 569 participants completed the survey at T1, with duplicates and missing cases removed. 377 participants had completed all three survey time points, with duplicates and missing cases removed.

PLWD – People living with dementia

For peer review only - http://bmjopen.bmj.com/site/about/guidelines.xhtml

For peer review only

For peer review only - http://bmjopen.bmj.com/site/about/guidelines.xhtml

Figure 1. Flow of participation in longitudinal survey

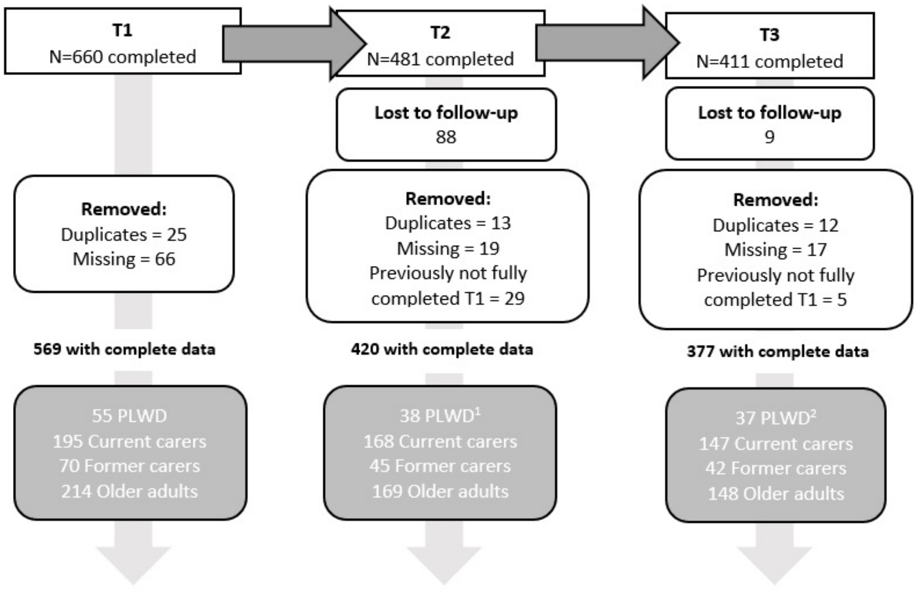

151x102mm (144 x 144 DPI)

Figure 2. Social support service usage pre-pandemic and at 3 survey time points

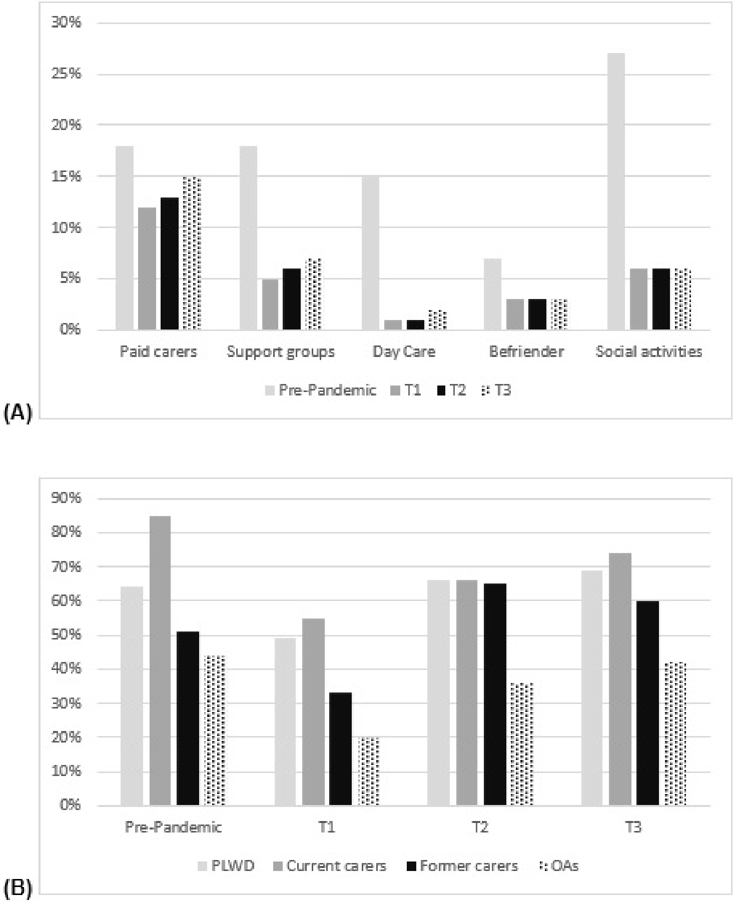

109x112mm (144 x 144 DPI)

Figure 3. Proportion of the total sample who scored above the cut offs for anxiety and depression at three time points

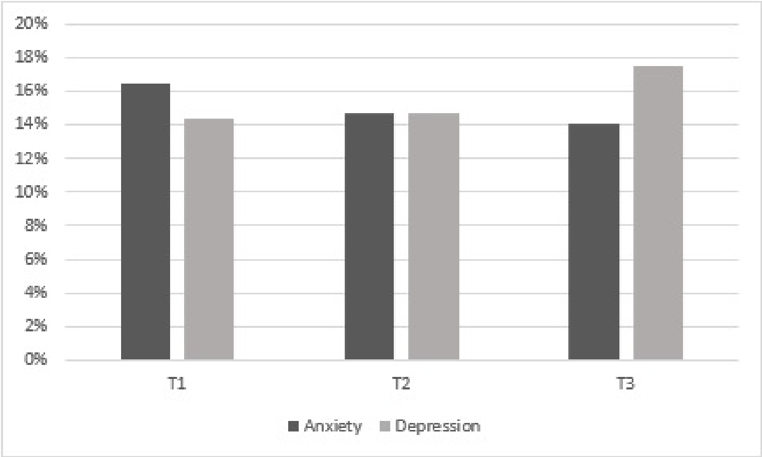

100x54mm (144 x 144 DPI)

Figure 4. Variations in anxiety, depression, and quality of life total scores at 3 time points

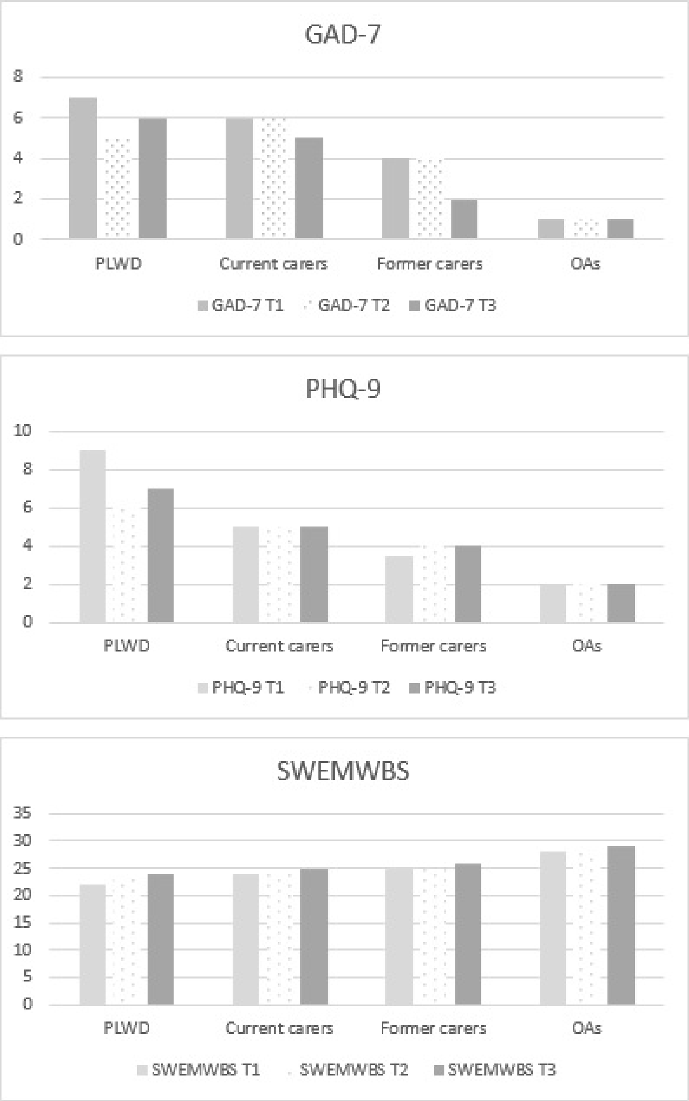

101x121mm (144 x 144 DPI)

BMJ Open

COVID-19-related social support service closures and mental well-being in older adults and those affected by dementia: A UK longitudinal survey

Journal:	*BMJ Open*
Manuscript ID	bmjopen-2020-045889.R1
Article Type:	Original research
Date Submitted by the Author:	15-Dec-2020
Complete List of Authors:	Giebel, Clarissa; University of Liverpool, Institute of Psychology, Health and Society<br>Pulford, Daniel; Lancashire Care NHS Foundation Trust<br>Cooper, Claudia; University College London<br>Lord, Kathryn; University of Bradford<br>Shenton, Justine; Sefton Older People's Forum<br>Cannon, Jacqueline; Lewy Body Society<br>Shaw, Lisa; University of Liverpool, Institute of Psychology, Health and Society<br>Tetlow, Hilary; SURF Liverpool<br>Limbert, Stan; NIHR ARC NWC<br>Callaghan, Steve; EQE Health<br>Whittington, Rosie; Me2U Care<br>Rogers, Carol; National Museums Liverpool<br>Komuravelli, Aravind; North West Boroughs Healthcare NHS Foundation Trust<br>Rajagopal, Manoj; Lancashire Care NHS Foundation Trust<br>Eley, Ruth; Liverpool DAA<br>Downs, Murna; University of Bradford, Bradford Dementia Group<br>Reilly, Siobhan; Lancaster University, Division of Health Research<br>Ward, Kym; The Brain Charity<br>Gaughan, Anna; TIDE<br>Butchard, Sarah; University of Liverpool, Institute of Psychology, Health and Society<br>Beresford, Jules; University of Bradford<br>Watkins, Caroline; University of Central Lancashire, Faculty of Health and Wellbeing<br>Bennett, Kate; University of Liverpool, Institute of Psychology, Health and Society<br>Gabbay, Mark
**Primary Subject Heading**:	Public health
Secondary Subject Heading:	Mental health
Keywords:	Dementia < NEUROLOGY, COVID-19, MENTAL HEALTH, Old age psychiatry < PSYCHIATRY

SCHOLARONE™
Manuscripts

I, the Submitting Author has the right to grant and does grant on behalf of all authors of the Work (as defined in the below author licence), an exclusive licence and/or a non-exclusive licence for contributions from authors who are: i) UK Crown employees; ii) where BMJ has agreed a CC-BY licence shall apply, and/or iii) in accordance with the terms applicable for US Federal Government officers or employees acting as part of their official duties; on a worldwide, perpetual, irrevocable, royalty-free basis to BMJ Publishing Group Ltd ("BMJ") its licensees and where the relevant Journal is co-owned by BMJ to the co-owners of the Journal, to publish the Work in this journal and any other BMJ products and to exploit all rights, as set out in our licence.

The Submitting Author accepts and understands that any supply made under these terms is made by BMJ to the Submitting Author unless you are acting as an employee on behalf of your employer or a postgraduate student of an affiliated institution which is paying any applicable article publishing charge ("APC") for Open Access articles. Where the Submitting Author wishes to make the Work available on an Open Access basis (and intends to pay the relevant APC), the terms of reuse of such Open Access shall be governed by a Creative Commons licence – details of these licences and which Creative Commons licence will apply to this Work are set out in our licence referred to above.

Other than as permitted in any relevant BMJ Author's Self Archiving Policies, I confirm this Work has not been accepted for publication elsewhere, is not being considered for publication elsewhere and does not duplicate material already published. I confirm all authors consent to publication of this Work and authorise the granting of this licence.

For peer review only

COVID-19-related social support service closures and mental well-being in older adults and those affected by dementia: A UK longitudinal survey

Clarissa Giebel[1,2,*], Daniel Pulford[3], Claudia Cooper[4], Kathryn Lord[5], Justine Shenton[6], Jacqueline Cannon[7], Lisa Shaw[8], Hilary Tetlow[9], Stan Limbert[2], Steve Callaghan[10], Rosie Whittington[11], Carol Rogers[12], Aravind Komuravelli[13], Manoj Rajagopal[3], Ruth Eley[14], Murna Downs[5], Siobhan Reilly[5], Kym Ward[15], Anna Gaughan[16], Sarah Butchard[17], Jules Beresford[5], Caroline Watkins[2,18], Kate Bennett[19], Mark Gabbay[1,2]

1 Department of Primary Care & Mental Health, University of Liverpool, UK
2 NIHR ARC NWC, Liverpool, UK
3 Lancashire and South Cumbria NHS Trust, UK
4 Division of Psychiatry, UCL, UK
5 Centre for Applied Dementia Studies, University of Bradford, UK
6 Sefton Older People's Forum, Sefton, UK
7 Lewy Body Society, Wigan, UK
8 Department of Modern Languages and Cultures, University of Liverpool, UK
9 SURF Liverpool, UK
10 EQE Health, Liverpool, UK
11 Me2U Care, Liverpool, UK
12 National Museums Liverpool, UK
13 North West Boroughs NHS Trust, Warrington, UK
14 Liverpool Dementia Action Alliance, UK
15 The Brain Charity, Liverpool, UK
16 TIDE, Liverpool, UK
17 Mersey Care NHS Trust, Liverpool, UK
18 Department of Health Research, UCLAN, UK
19 School of Psychology, University of Liverpool, UK

**Correspondence should be addressed to:* Clarissa Giebel, Waterhouse Building, University of Liverpool, Liverpool, L69 3GL. Email: Clarissa.giebel@liverpool.ac.uk

For peer review only

For peer review only - http://bmjopen.bmj.com/site/about/guidelines.xhtml

Abstract

Background: The COVID-19 pandemic has had a major impact on delivery of social support services. This might be expected to particularly affect older adults and people living with dementia (PLWD), and to reduce their wellbeing.

Aims: To explore how social support service use by older adults, carers, and PLWD, and their mental wellbeing changed over the first three months since the pandemic outbreak.

Methods: Unpaid dementia carers, PLWD, and older adults took part in a longitudinal online or telephone survey collected in April-May 2020, and at two subsequent time points 6 and 12 weeks after baseline. Participants were asked about their social support service usage in a typical week prior to the pandemic (at baseline), and in the past week at each of the three time points. They also completed measures of levels of depression, anxiety and mental well-being.

Results: 377 participants had complete data at all three time points. Social support service usage dropped shortly after lockdown measures were imposed at timepoint 1 (T1), to then increase again by T3. The access to paid care was least affected by COVID-19. Cases of anxiety dropped significantly across the study period, whilst cases of depression rose. Well-being increased significantly for older adults and PLWD from T1 to T3.

Conclusions: Access to social support services has been significantly affected by the pandemic, which is starting to recover slowly. With mental well-being differently affected across groups, support needs to be put in place to maintain better well-being across those vulnerable groups during the ongoing pandemic.

For peer review only

Strengths and limitations of this study

- Data on social support service usage and mental health were collected at three time points in the early stages of the pandemic
- 63% of participants completed all three survey time points
- The survey was co-produced with people affected by dementia
- The study was mostly completed by people from a White ethnic background, and lacks ethnic minority representation despite actively approaching community groups
- Some people with dementia completed the survey, who may have had difficulties in recalling the services they received pre-pandemic

For peer review only

For peer review only - http://bmjopen.bmj.com/site/about/guidelines.xhtml

Introduction

In the UK, 11.9 million people are aged 65 and over [1], with over 850,000 living with dementia [2]. Social support services, including day care centres, support groups, paid home carers, and community activities, such as singing or arts groups, are important for maintaining a good quality of life for older people and people living with dementia (PLWD) [3-4]. In view of an ageing population and increasing numbers of PLWD, easily accessible services are crucial to support people socially, as well as with their care needs.

The COVID-19 pandemic has affected these social support services significantly. Social isolation as a result of social distancing, lockdowns, and shielding is a huge concern for older people across the globe since the beginning of the pandemic [5-6], with similar issues highlighted early for PLWD [7]. However, there is still a dearth of evidence on the mental well-being and access to care for those in need.

Specifically, in the UK, a nationwide three-month lockdown was imposed on the 23rd of March. Older people were over-represented in the group who were clinically extremely vulnerable, that is at greatest risk of severe illness from COVID-19, who were asked to shield by the government until early August. All adults aged 70+ were classified as being at least moderate risk of severe illness from COVID-19 [8]. During the most restrictive, earlier period of lockdown, people were advised to only go outside once a day for essential food shopping, pharmacy visits, or to exercise. Non-essential shops were closed, and only started reopening in July. With additional social distancing for the general population, and use of personal protective equipment (PPE) for the health and social care taskforce in place, these measures significantly impact the social support services that PLWD, carers, and older adults could receive. Recent qualitative evidence has highlighted how PLWD and unpaid carers have faced a sudden crisis in terms of accessing social support services since the pandemic [9], and have faced difficult decisions whether to continue or discontinue paid carers entering the home of the PLWD, for fear of potential virus transmission [10]. Whilst these qualitative accounts provide rich information on the experiences of having accessed (or failed to access) social support services during the pandemic, there appears to be no empirical evidence to date quantifying those experiences and linking these with mental well-being.

The aim of this exploratory study was to explore the impacts of COVID-19 on social support service closures and longitudinal changes in the mental illness and well-being of older adults, PLWD, and unpaid carers.

Methods

Participants and recruitment

We recruited UK residents who were aged 18+. PLWD were eligible to take part if they had a diagnosis of dementia. Unpaid carers were eligible to take part if they were (current carers) or had been caring for a relative or friend with dementia (former carers). Older adults were eligible to take part if they were aged 65 years or older.

Participants were recruited via different social support services third sector organisations, such as peer support group organisations, carer networks, cultural dementia training programme organisations, and national dementia subtype specific organisations, and by contacting people on their email circulation lists, via newsletters and social media accounts. We also directly contacted people who were accessing regular services, such as support groups or older people fora, via telephone. This ensured that people without internet access were able to participate in this research. We also utilised *Join Dementia Research*, a UK-wide

For peer review only - http://bmjopen.bmj.com/site/about/guidelines.xhtml

national online register of PLWD, carers, older adults, and health volunteers who are interested in taking part in dementia and ageing research.

Ethical approval was obtained from the University of Liverpool prior to study begin [Ref: 7626].

Data collection

The study was completed at three time points (T1, T2, T3), 5 and 6 weeks apart, respectively. Participants could complete the survey either online or over the phone with a research team member who entered their details into the online survey on their behalf. Participants from T1 (baseline) were followed-up with the same mental well-being questionnaires at T2 and T3 and were followed-up either by telephone or email, depending on how they completed T1 survey. T1 ran from 17th April to 15th May (+/- 3 days). T2 ran from 29th May to 26th June (+/- 3 days). T3 ran from 10th July to 7th August (+/- 3 days).

Variables and tools

At T1, participants were asked about their background characteristics (including age, gender, ethnicity, postcode, living situation, type of dementia (if applicable), and employment). Postcode data were collected to generate an Index of Multiple Deprivation (IMD) quintile. IMD provides a measure of neighbourhood deprivation, taking into account income, education, crime, and health, amongst others. Quintile 1 indicates least deprived neighbourhoods, with quintile 5 indicating the most deprived neighbourhoods.

Service usage was measured by asking about pre-pandemic and current receipt of different social support services (including paid carers, support groups, befrienders, day care centres, respite, meal deliveries, transport, social activities, clinical mental health support, and clinical physical support) and equipment, such as hand rails or shower seats, as well as the weekly total hours of social support services. Pre-pandemic service usage was defined as use of social support services in a typical week before the pandemic.

Participants were also asked to complete the Personalised Health Questionnaire 9 (PHQ-9) [11] for levels of depression, the Generalised Anxiety Disorder 7 (GAD-7) [12] for levels of anxiety, and the Short Warwick-Edinburgh Mental Well-Being Scale [13] (SWEMWBS) for quality of life. Higher scores on the PHQ-9, GAD-7, and the SWEMWBS indicated higher levels of depression, anxiety, and quality of life, respectively. We categorised participants who scored of 10 or more on the PHQ-9 as 'depressed' and on the GAD-7 as 'anxious', as based on previous extensive research, indicating that these cut offs indicate general anxiety disorder and depression, respectively [14]. At T2 and T3, participants were asked again about their current levels of social support service receipt, weekly hours of support, equipment, as well as the PHQ-9, GAD-7, and SWEMWBS.

Data analysis

Data were analysed using SPSS 25, and the significance level was set at $p<0.05$. Participant demographic characteristics and social support service usage and mental well-being variables were analysed using frequency analysis. Chi-square tests were used to assess variations in the proportions of participants who were categorised as 'depressed' or 'anxious'. Repeated measures ANOVAs with Greenhouse Geisser posthoc correction were used to analyse differences between T1, T2, and T3 in GAD-7 total, PHQ-9 total, and SWEMWBS total scores. For this analysis, only participants with complete GAD-7, PHQ-9, and SWEMWBS were included (n=377). Paired samples t-tests were employed to compare the means of GAD-7, PHQ-9, and SWEMWBS at T1 between those who completed all three time points (n=377)

For peer review only - http://bmjopen.bmj.com/site/about/guidelines.xhtml

and those who dropped out after T1 or T2 (n=192). Bivariate correlation analysis was employed to assess whether changes in weekly social support service usage from pre-pandemic to T1 were associated with changes in GAD-7, PHQ-9, and SWEMWBS between T1 and T3.

Public involvement
Unpaid carers and a PLWD were involved as equal team members in all aspects of the study – from conceptualisation and design through to analysis and dissemination.

Results

Survey completion
Figure 1 outlines the participant flow and completion rates in further detail. 569 participants completed the survey at T1 (61 PLWD; 219 current carers; 66 former carers; 223 older adults). 420 participants completed the survey at T2 (38 PLWD; 168 current carers; 45 former carers; 169 older adults). 377 participants completed all three waves of the survey (37 PLWD; 147 current carers; 42 former carers; 148 older adults).

Participant characteristics
Table 1 shows the demographic characteristics of those who completed T1 and those that completed all three survey time points, by subgroup. For those who completed all three time points, carers and older adults were mostly female (59-82%), whilst the majority of PLWD were male (62%). The majority of participants were from a White ethnic background (95-99%) and lived with someone else (61-88%), with current carers having the highest proportion of living with someone else. The majority of participants across all four groups lived in less deprived neighbourhoods (Quintiles 1 and 2) (52-61%). Thirty-seven PLWD took part in all three time points; the most common diagnostic subtype was Alzheimer's disease.

[Figure 1 and Table 1]

Social support service and activities usage
Participants had accessed a range of social support services pre-pandemic, including day care centres, support groups, meal deliveries, respite, and paid carers. Figure 2 (A) shows the proportion of participants of the total sample (n=377) who reported accessing paid carers, support groups, day care, befrienders, and social activities prior to the pandemic, and at T1, T2, and T3. These were the most commonly used types of social support services prior to the pandemic. Social support services usage had dropped since the pandemic outbreak. Pre-pandemic, 27% of participants accessed social activities in the community, which dropped to 6% at T1, T2, and T3. Paid care saw the smallest change – with 17% having accessed paid carers pre-pandemic, dropping to 12% at T1 and increasing slightly again to 15% at T3. Day care saw the largest drop, with only 1-2 % receiving day care since the outbreak, compared to 15% previously.

Figure 2 (B) shows the proportion of participants by group who have received any form of social support services pre-pandemic and at all three survey time points. Pre-pandemic, 90% of current carers had received social support of any form, with between 45% and 50% of former carers and older adults having received some support. This decreased at T1 for all groups to between 20% (older adults) to 55% (current carers) receiving some type of support. Through T2 and T3, an upward trend emerged with more participants gaining access to some services again, with levels for PLWD and former carers being higher at T3 than at pre-pandemic levels.

[Figure 2]

For peer review only - http://bmjopen.bmj.com/site/about/guidelines.xhtml

Mental well-being

Figure 3 shows the proportion of participants across the total sample (n=377) who were categorised and identified as anxious and depressed across all three time points. For anxiety, we noted a downward trend in number of cases from T1 (16.5%) to T3 (14.1%). The proportion of participants with anxiety was significantly lower at T2 (x^2=186.399, p<0.001) and T3 (x^2=136.562, p<0.001) compared to T1. For depression, we noted an upward trend in cases, as indicated based on their cut off on the PHQ-9, from T1 (14.4%) to T3 (17.5%). The proportion of participants with depression was significantly higher at T2 (x^2= 176.248, p<0.001) and T3 (x^2=158.031, p<0.001) compared to T1.

Figure 4 shows the median of the GAD-7, PHQ-9, and SWEMWBS total scores by group over time for those who completed all three survey time points. Based on the median scores, levels of anxiety and depression appear to decrease from T1 to T3, whilst quality of life increases from T1 to T3.

Paired samples t-test showed that there were no significant differences in means of GAD-7 (p=.468), PHQ-9 (p=.183), and SWEMWBS (p=.332) at T1 between those who completed all three survey time points and those who dropped out after T1 or T2.

For anxiety, repeated-measures ANOVAs with Greenhouse-Geisser posthoc correction showed that GAD-7 total scores did not vary significantly from T1 to T3 for PLWD [F(1.856, 64.962)= 1.429, p= 0.247]or among current carers [F(1.898, 277.063)= 1.938, p=0.148], former carers[F(1.801, 68.419)=.139, p=.139], or older adults [F(1.924, 286.727)= 2.688, p=.0072], based on those participants who completed in all three survey time points.

For depression, repeated-measures ANOVAs showed that PHQ-9 total scores did not significantly vary from T1 to T3 for PLWD [F(1.896, 66.370)= 1.461, p=0.240], current carers [F(1.900, 277.453)= .639, p=0.521], former carers [F(1.677, 68.419)= .024, p=0.960], or older adults [F(1.889, 281.414)= .857, p=0.420].

For well-being, repeated-measures ANOVAs showed that SWEMWBS total scores significantly increased from T1 to T3 for PLWD [F(1.726, 60.423)= 5.412, p<0.05 [Mean(SD) T1-T3= 22.1(6.4); 24.3(5.6); 24.4(5.6)]] and older adults [F(1.804, 268.807)= 3.632, p<0.05 [Mean(SD) T1-T3= 27.5(5.1); 28.0(5.1); 28.3(4.9)]]. However there were no significant changes among current [F(1.982,289.325)=2.185, p=0.115] or former carers [F(1.728, 63.725)= .268, p=0.733].

[Figure 3 and 4]

Social support and mental health

Bivariate correlation analyses showed no significant associations between variations in social support service hours between pre-pandemic and at T1 and changes in SWEMWBS (p=.332), GAD-7 (p=.310), and PHQ-9 (p=.351) between T1 and T3 for those who completed all three survey time points.

Discussion

This is one of the first studies to show that social support service usage in dementia and ageing reduced significantly compared to pre-pandemic levels, whilst slowly rising in the months post-nationwide lockdown. In addition, we also show that cases of anxiety reduced whilst cases of depression increased in the months since lockdown, with quality of life significantly increasing for PLWD and older adults only.

Social support service usage for PLWD, unpaid carers, and older adults has seen a significant decrease since the COVID-19 pandemic, leaving many people suddenly without vital support – ranging from day care centres to respite to support groups. In the months following the nationwide lockdown, usage has gradually increased again but varied among

For peer review only - http://bmjopen.bmj.com/site/about/guidelines.xhtml

providers and type of support. With public health restrictions still remaining in place however during that period, including social distancing, shielding and thus inability to meet members of different households, such support is most likely to be implemented via digital technologies. Considering that in our sample 94% of participants completed the survey online rather than the telephone option, nearly all participants had access to the internet. However, many older adults and PLWD are less likely to be digitally literate [15], making it difficult for all people to access services equally. This has already been an issue pre-COVID-19 [16], suggesting that the pandemic has further exacerbated potential inequalities in access and thus further isolated people who would benefit from social support the most.

One type of support which has been affected the least by the pandemic has been paid home care. Receiving paid home care enables PLWD and older adults to stay at home independently for longer – as people wish to avoid entering a care home and stay in their familiar environment [17]. Whilst there was a reduction in paid home care usage compared to pre-pandemic levels, overall paid home carers were utilised the most. A qualitative exploration into decision-making for whether or not to continue paid home care during the pandemic has shown that many unpaid carers were afraid of having paid carers enter the home (often with inadequate PPE) for risk of potential virus transmission [10]. Other unpaid carers however felt unable to cope without the support, or indeed accepted the potential risks, and continued paid home care. There is also a notable difference between social care provision (which is paid home care) and third sector care provision (which involves support groups and social activities for example). The third sector relies on volunteers providing services, and has also suffered during the pandemic, whereas the social care sector is financially supported by the government. Therefore, the ability to receive home care might not have been affected to the same extent as accessing support groups for instance. Another potential reason for variations in usage between activity types is that home care involves someone from the outside entering someone's home. In contrast, day care centres, respite care, and social activities involve older adults and PLWD going outside to larger social gatherings. Due to public health restrictions, these have been temporarily rendered largely, if not completely, impossible to take place in their original face-to-face formats. As numbers of infections rise again, these restrictions are being strengthened and re-imposed with large fines possible for those transgressing them.

Anxiety, depression, and well-being changed over the course of the study period. Over 12 weeks, cases of anxiety across the total sample dropped, whilst cases of depression increased significantly. However, when exploring levels of anxiety and depression within groups, no significant changes were noted, which is likely to have been due to small and varied sample sizes for each subgroup. This may be because people who were very depressed or anxious might not have continued the follow-up surveys. It is also possible though that participants felt more connected over time, particularly considering again that the majority of participants completed the survey online and thus were able to participate in remote services, where these existed. Recent evidence from Spain showed how older adults were less likely to suffer from psychological distress as a result of the pandemic than people aged below 60 [18]. Nevertheless, overall the pandemic is having a heightened impact on the mental health of the general population [19-20]. Engaging in social activities can be one avenue to help maintain good mental health [21]. Considering that reductions in social engagement both before and after a dementia diagnosis are common [22], enabling continued engagement throughout the pandemic is important to support PLWD, carers, and older adults adequately. This is corroborated by evidence from the baseline survey showing that reductions in social support usage were linked to mental well-being [23]. It is possible that for this study, merging groups of older adults, PLWD, and carers resulted in no significant associations, as each group was differently affected, as indicated by looking at changes of mental well-being for each group across the 12 weeks.

For peer review only - http://bmjopen.bmj.com/site/about/guidelines.xhtml

There were some limitations to our exploratory study. Whilst benefitting from a large sample size and good retention rate over a relatively short time period of 12 weeks, the majority of participants had internet access and were thus also able to join in remote social support. Although we actively approached older adults, PLWD, and carers via phone through recruiting organisations, only some people took part over the phone. It is likely however that those people without internet access have been even more isolated through the pandemic, with potentially severe mental health needs, which we have only captured a snapshot of. This also links to the fact that there are likely to be longer-term effects on mental well-being, with our survey only providing a snap shot of the first few months since the start of the pandemic. Equally, our survey did not include pre-pandemic levels of mental well-being (anxiety, depression, and quality of life), which would have provided additional insight into changes in mental well-being. However, due to the unforeseen circumstance of the pandemic, it was not feasible to collect these data. We only enquired about weekly hours of total social support usage, and not for each specific type of activity. Some participants might have accessed, for example, paid home care, but only for two hours as opposed to others who might have received 40 hours a week. We are thus unable to state in detail how the pandemic has affected the level of each different type of support, but instead we provide a more general overview of activities and general service usage variations since the nationwide lockdown, which to existing knowledge has not been captured elsewhere.

Conclusions

The pandemic is having a sudden and severe long-term impact on social support service usage for older adults and people affected by dementia, which sees somewhat of a limited increase in usage over the first few months since nationwide lockdown. Whilst it appears that some services have started providing remote support, not everyone will be able to access these, leaving many people without much needed support. Future research needs to assess how older adults and people affected by dementia are accessing social support services in the time of COVID-19, with clearer support for people to access any format of services – either face-to-face or remotely. Considering that the pandemic is going to continue for the foreseeable future, the mental health of older adults and those affected by dementia needs to be closely monitored, particularly when more stringent public health measures are put in place again.

For peer review only - http://bmjopen.bmj.com/site/about/guidelines.xhtml

Conflicts of interest
None.

Acknowledgements
This study would not have been possible without our participants, thank you very much. We also wish to thank Join Dementia Research for helping us recruit to our study.

Funding
This research is supported by a grant awarded to the authors by the University of Liverpool COVID-19 Strategic Research Fund in 2020. This is also independent research funded by the National Institute for Health Research Applied Research Collaboration North West Coast (ARC NWC). The views expressed in this publication are those of the author(s) and not necessarily those of the National Institute for Health Research or the Department of Health and Social Care. The University of Bradford QR Research Fund also supported part of this study. There are no grant numbers.

Data availability statement
Data may be obtained from a third party and are not publicly available.

Author contributions
CG managed data collection, conducted analysis, and wrote the manuscript. JC, JS, and DP collected data over the phone. DP managed the data. All authors (CG, DP, CC, KL, JS, JC, LS, HT, SL, SC, RW, CR, AK, MR, RE, MD, SR, KW, AG, SB, JB, CW, KB, MG) contributed to designing the survey, interpreting the findings, and reading drafts of the manuscript. All authors approved the final manuscript. Emma Riley helped with recruitment by emailing eligible participants via the House of Memories networks and we wish to thank her for her support with this.

For peer review only

Figure Captions

Figure 1. Flow of participation in longitudinal survey

NOTE. The top boxes indicate how many people completed each survey time point. After having removed (1) duplicates (people who completed the survey twice), (2) missing cases (where participants had not completed the PHQ-9, GAD-7, and the SWEMWBS or had missing ID codes at T2 and T3), and (3) losses to follow-up (those that had either completed T1 or T1 and T2 only), and (4) incomplete data at T1 yet data at T2 or T3, 377 cases remained in total. Grey boxes indicate the breakdown by subgroup.

[1] Follow-up completion by subgroup by percentage at T2 and T3 compared to T1: PLWD 69%(T2), 67%(T3); Current Carers 86%(T2), 75%(T3); Former carers 64%(T2), 60%(T3); Older adults 79%(T2), 69%(T3).

Figure 2. Social support service usage pre-pandemic and at 3 survey time points

Note. (A) Service usage for the total sample (N=377) in proportion of participants at four different time points for some of the most frequently used support services. (B) Proportion of participants within each group at four different timepoints (pre-pandemic, T1, T2, T3) having received any form of social support.

Figure 3. Proportion of the total sample who scored above the cut offs for anxiety and depression at three time points

NOTE. T = Time point

The graph shows the proportion of participants from the total sample who completed all three surveys (n=377) and scored above the cut off on the GAD-7 and PHQ-9 for anxiety and depression, respectively.

Figure 4. Variations in anxiety, depression, and quality of life total scores at 3 time points

NOTE. Figures show the median total score at each time point (T1, T2, T3) for each subgroup – for anxiety (GAD-7), depression (PHQ-9), and quality of life (SWEMWBS).

For peer review only

References

For peer review only - http://bmjopen.bmj.com/site/about/guidelines.xhtml

[1] Office for National Statistics (ONS). *Living longer: is age 70 the new age 65?* London, England 2019: ONS.

[2] Alzheimer's Society. *Dementia UK: An Update*. London, England: Alzheimer's Society 2014.

[3] Skingley A, Martin A, Clift S. The contribution of community singing groups to the well-being of older people: participant perspectives from the United Kingdom. Journal of Applied Gerontology 2016;35(12):1302-1324.

[4] Tretteteig S, Vatne S, Mork Rokstad AM. The influence of day care centres designed for people with dementia on family caregivers – a qualitative study. BMC Geriatrics 2017;17(5).

[5] Armitage R, Nellums LB. COVID-19 and the consequences of isolating elderly. LANCET Public Health 2020; 5(5):doi: 10.1016/S2468-2667(20)30061-X

[6] Berg-Weger M, Morley JE. Loneliness and social isolation in older adults during the COVID-19 pandemic: implications for gerontological social work. Journal of Nutrition, Health & Ageing 2020;24(5):456-458.

[7] Brown EE, Kumr S, Rajji TK, et al. Anticipating and mitigating the impact of the COVID-19 pandemic on Alzheimer's disease and related dementias. American Journal of Geriatric Psychiatry 2020;28(7):712-721.

[8] Public Health England (PHE). *Guidance on shielding and protecting people who are clinically extremely vulnerable from COVID-19.* London, England 2020: PHE. Last accessed 22nd September 2020: https://www.gov.uk/government/publications/guidance-on-shielding-and-protecting-extremely-vulnerable-persons-from-covid-19/guidance-on-shielding-and-protecting-extremely-vulnerable-persons-from-covid-19#definition

[9] Giebel C, Cannon J, Hanna K, et al. Impact of Covid-19 related social support service closures on people with dementia and unpaid carers: A qualitative study. Aging & Mental Health 2020a; doi: 10.1080/13607863.2020.1822292

[10] Giebel C, Hanna K, Cannon J, et al. Decision-making for receiving paid home care for dementia in the time of COVID-19: A qualitative study. BMC Geriatrics 2020b; doi: 10.1186/s12877-020-01719-0

[11] Kroenke K, Spitzer RL, Williams JB. The PHQ-9: validity of a brief depression severity measure. Journal of General Internal Medicine 2001;16(9):606-613.

[12] Spitzer RL, Kroenke K, Williams JB, Loewe B. A brief measure for assessing generalized anxiety disorder: the GAD-7. Archives of Internal Medicine 2006;166(10):1092-1097.

[13] Haver A, Akerjordet K, Caputi P, et al. Measuring mental well-being: A validation of the Short Warwick-Edinburgh Mental Well-Being Scale in Norwegian and Swedish. Scandinavian Journal of Public Health 2015;43(7):721-727.

[14] Smith, B.W., Epstein, E.E., Oritz, J.A., Christopher, P.K., & Tooley, E.M. (2013). The Foundations of Resilience: What are the critical resources for bouncing back from stress? In Prince-Embury, S. & Saklofske, D.H. (Eds.), *Resilience in children, adolescents, and adults: Translating research into practice*, The Springer series on human exceptionality (pp. 167-187). New York, NY: Springer.

For peer review only - http://bmjopen.bmj.com/site/about/guidelines.xhtml

[15] Hill R, Betts LR, Gardner SE. Older adults' experiences and perceptions of digital technology: (Dis)empowerment, wellbeing, and inclusion. Computers in Human Behavior 2015;48: 415-423.

[16] Stephan A, Bieber A, Hopper L, et al. Barriers and facilitators to the access to and use of formal dementia care: findings of a focus group study with people with dementia, informal carers and health and social care professionals in eight European countries. BMC Health Services Research 2018; 131: doi: 10.1186/s12877-018-0816-1

[17] Rapaport P, Burton A, Leverton M, et al. "I just keep thinking that I don't want to rely on people." A qualitative study of how people living with dementia achieve and maintain independence at home: stakeholder perspectives BMC Geriatrics 2020; doi: 10.1186/s12877-019-1406-6

[18] Garcia-Portilla P, de la Fuente Tomas L, Bobes-Bascaran T, et al. Are older adults also at higher psychological risk from COVID-19? Aging & Mental Health 2020; doi: 10.1080/13607863.2020.1805723

[19] Fancourt D, Steptoe A, Bu F. Trajectories of depression and anxiety during enforced isolation due to COVID-10: longitudinal analyses of 59,318 adults in the UK with and without diagnosed mental illness. medRxiv preprint 2020; doi: 10.1101/2020.06.03.20120923

[20] Iob E, Steptoe A, Fancourt D. Abuse, self-harm and suicidal ideation in the UK during the COVID-19 pandemic. British Journal of Psychiatry 2020; doi: 10.1192/bjp.2020.130

[21] Holtfreter K, Reisig MD, Turanovic JJ. Depression and infrequent participation in social activities among older adults: the moderating role of high-quality familial ties. Aging & Mental Health 2017;21(4): 10.1080/13607863.2015.1099036

[22] Hackett RA, Steptoe A, Cadar D, Fancourt D. Social engagement before and after dementia diagnosis in the English Longitudinal Study of Ageing. PLOS One 2019; doi: 10.1371/journal.pone.0220195

[23] Giebel C, Lord K, Cooper C, et al. A UK survey of COVID-19 related social support service closures and the effects on older people, people with dementia, and carers. International Journal of Geriatric Psychiatry 2020c; doi: 10.1002/gps.5434

For peer review only

Table 1. Participant characteristics of those completing T1 survey and those completing all three survey time points

	T1 (n=569)				T1, 2 and 3 (n=377)			
	PLWD (n=61)	Current carers (n=219)	Former carers (n=66)	Older adults (n=223)	PLWD (n=37)	Current carers (n=149)	Former carers (n=39)	Older adults (n=152)
**N(%)**								
**Gender**								
**Female**	27 (44.3)	168 (77.1)	55 (83.3)	137 (61.7)	14 (37.8)	118 (79.7)	32 (82.1)	90 (59.2)
**Male**	34 (55.7)	50 (22.9)	11 (16.7)	85 (38.3)	23 (62.2)	30 (20.3)	7 (17.9)	62 (40.8)
**Ethnicity**								
**White**	58 (96.7)	211 (96.3)	65 (98.5)	216 (98.2)	35 (94.6)	143 (96.0)	38 (97.4)	148 (98.7)
**Other**	2 (3.4)	8 (3.7)	1 (1.5)	4 (1.9)	2 (5.4)	6 (4.0)	1 (2.6)	2 (1.3)
**Living situation**								
**Alone**	13 (21.3)	33 (15.1)	17 (26.2)	79 (35.6)	8 (21.6)	18 (12.2)	11 (28.9)	59 (39.1)
**With someone**	48 (78.7)	185 (84.9)	48 (73.8)	143 (64.4)	29 (78.4)	130 (87.8)	27 (71.1)	92 (60.9)
**IMD Quintile**								
**1**	12 (23.1)	54 (32.1)	10 (19.2)	61 (33.5)	5 (16.1)	35 (31.0)	6 (19.4)	39 (31.7)
**2**	16 (30.8)	50 (29.8)	20 (38.5)	44 (24.2)	11 (35.5)	34 (30.1)	11 (35.5)	29 (23.6)
**3**	10 (19.2)	32 (19.0)	14 (26.9)	37 (20.3)	6 (19.4)	20 (17.7)	9 (29.0)	26 (21.1)
**4**	10 (19.2)	14 (8.3)	5 (9.6)	26 (14.3)	5 (16.1)	11 (9.7)	4 (12.9)	18 (14.6)
**5**	4 (7.7)	18 (10.7)	3 (5.8)	14 (7.7)	4 (12.9)	13 (11.5)	1 (3.2)	11 (8.9)
**Type of dementia**								
**Alzheimer's**	20 (32.8)	100 (46.5)	6 (23.1)		14 (37.8)	75 (50.7)	2 (20.0)	
**Mixed**	13 (21.3)	49 (22.8)	7 (26.9)		6 (16.2)	34 (23.0)	4 (40.0)	
**Vascular**	11 (18.0)	27 (12.6)	4 (15.4)		8 (21.6)	18 (12.2)	3 (30.0)	
**Other**	17 (27.9)	39 (18.1)	9 (34.5)		9 (24.3)	21 (14.1)	1 (10.0)	
**Mean (SD), [Range]**								
**Age**	70 (+/-10), [45-88]	61 (+/-13), [23-89]	64 (+/-14), [22-95]	72 (+/-6), [65-90]	72 (+/-10), [50-88]	62 (+/-13), [23-89]	65 (+/-13), [22-95]	73 (+/-6), [65-90]
**Years of education**	15 (+/-4), [4-25]	16 (+/-4), [6-28]	17 (+/-4), [10-29]	17 (+/-4), [7-25]	13 (+/-4), [4-20]	16 (+/-4), [6-28]	16 (+/-4), [10-29]	16 (+/-4), [7-24]

NOTE. 569 participants completed the survey at T1, with duplicates and missing cases removed. 377 participants had completed all three survey time points, with duplicates and missing cases removed.

PLWD – People living with dementia

For peer review only - http://bmjopen.bmj.com/site/about/guidelines.xhtml

For peer review only

For peer review only - http://bmjopen.bmj.com/site/about/guidelines.xhtml

Figure 1. Flow of participation in longitudinal survey

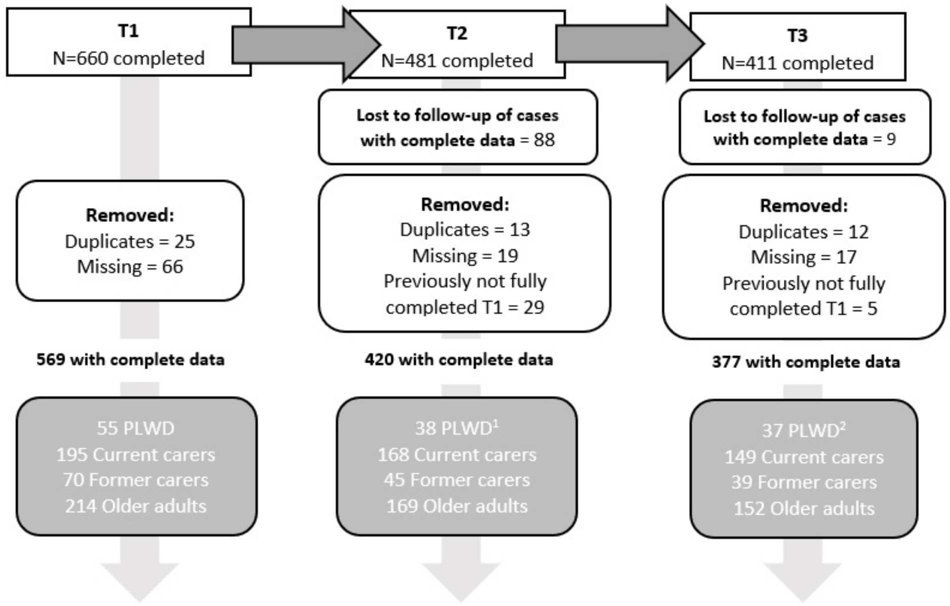

147x102mm (144 x 144 DPI)

Figure 2. Social support service usage pre-pandemic and at 3 survey time points

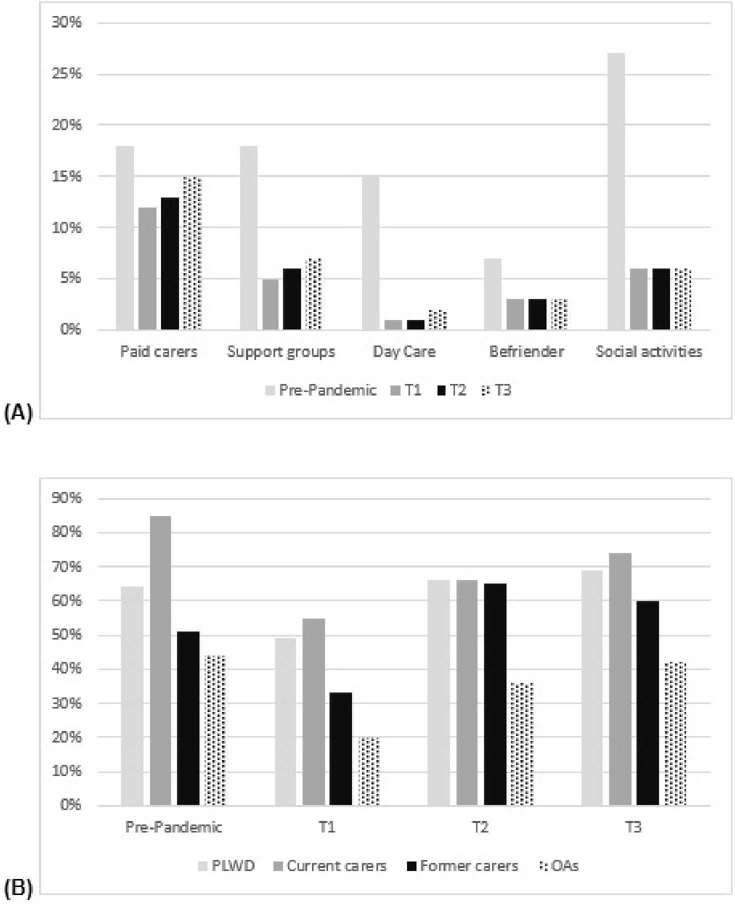

109x112mm (144 x 144 DPI)

Figure 3. Proportion of the total sample who scored above the cut offs for anxiety and depression at three time points

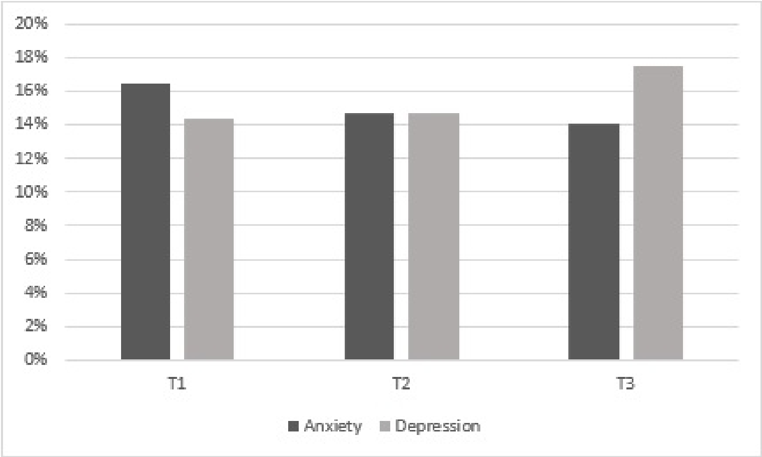

100x54mm (144 x 144 DPI)

Figure 4. Variations in anxiety, depression, and quality of life total scores at 3 time points

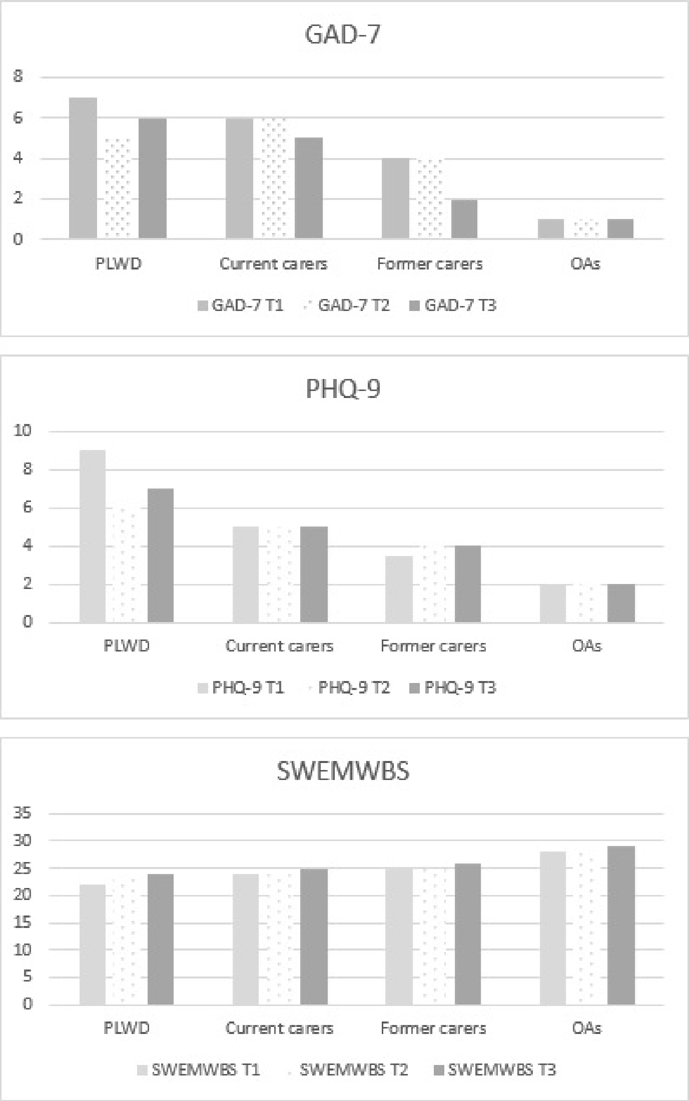

101x121mm (144 x 144 DPI)

STROBE Statement—checklist of items that should be included in reports of observational studies

	Item No	Page	Recommendation
**Title and abstract**	1	1 & 2	(*a*) Indicate the study's design with a commonly used term in the title or the abstract
		1 & 2	(*b*) Provide in the abstract an informative and balanced summary of what was done and what was found
**Introduction**			
Background/rationale	2	4	Explain the scientific background and rationale for the investigation being reported
Objectives	3	4	State specific objectives, including any prespecified hypotheses
**Methods**			
Study design	4	4-5	Present key elements of study design early in the paper
Setting	5	4-5	Describe the setting, locations, and relevant dates, including periods of recruitment, exposure, follow-up, and data collection
Participants	6	4-5	(*a*) *Cohort study*—Give the eligibility criteria, and the sources and methods of selection of participants. Describe methods of follow-up<br>*Case-control study*—Give the eligibility criteria, and the sources and methods of case ascertainment and control selection. Give the rationale for the choice of cases and controls<br>*Cross-sectional study*—Give the eligibility criteria, and the sources and methods of selection of participants
		4-5	(*b*) *Cohort study*—For matched studies, give matching criteria and number of exposed and unexposed<br>*Case-control study*—For matched studies, give matching criteria and the number of controls per case
Variables	7	5	Clearly define all outcomes, exposures, predictors, potential confounders, and effect modifiers. Give diagnostic criteria, if applicable
Data sources/ measurement	8*	5	For each variable of interest, give sources of data and details of methods of assessment (measurement). Describe comparability of assessment methods if there is more than one group
Bias	9	n/a	Describe any efforts to address potential sources of bias
Study size	10	6	Explain how the study size was arrived at
Quantitative variables	11	5	Explain how quantitative variables were handled in the analyses. If applicable, describe which groupings were chosen and why
Statistical methods	12	5	(*a*) Describe all statistical methods, including those used to control for confounding
		5	(*b*) Describe any methods used to examine subgroups and interactions
			(*c*) Explain how missing data were addressed
			(*d*) *Cohort study*—If applicable, explain how loss to follow-up was addressed<br>*Case-control study*—If applicable, explain how matching of cases and controls was addressed<br>*Cross-sectional study*—If applicable, describe analytical methods taking account of sampling strategy
			(*e*) Describe any sensitivity analyses

For peer review only - http://bmjopen.bmj.com/site/about/guidelines.xhtml

Continued on next page

For peer review only

For peer review only - http://bmjopen.bmj.com/site/about/guidelines.xhtml

Results			Page
Participants	13*	(a) Report numbers of individuals at each stage of study—eg numbers potentially eligible, examined for eligibility, confirmed eligible, included in the study, completing follow-up, and analysed	6
		(b) Give reasons for non-participation at each stage	
		(c) Consider use of a flow diagram	Figure 1
Descriptive data	14*	(a) Give characteristics of study participants (eg demographic, clinical, social) and information on exposures and potential confounders	6
		(b) Indicate number of participants with missing data for each variable of interest	6
		(c) *Cohort study*—Summarise follow-up time (eg, average and total amount)	
Outcome data	15*	*Cohort study*—Report numbers of outcome events or summary measures over time	*6*
		Case-control study—Report numbers in each exposure category, or summary measures of exposure	
		Cross-sectional study—Report numbers of outcome events or summary measures	
Main results	16	(*a*) Give unadjusted estimates and, if applicable, confounder-adjusted estimates and their precision (eg, 95% confidence interval). Make clear which confounders were adjusted for and why they were included	6-7
		(*b*) Report category boundaries when continuous variables were categorized	6-7
		(*c*) If relevant, consider translating estimates of relative risk into absolute risk for a meaningful time period	
Other analyses	17	Report other analyses done—eg analyses of subgroups and interactions, and sensitivity analyses	6-7
**Discussion**			
Key results	18	Summarise key results with reference to study objectives	7
Limitations	19	Discuss limitations of the study, taking into account sources of potential bias or imprecision. Discuss both direction and magnitude of any potential bias	8
Interpretation	20	Give a cautious overall interpretation of results considering objectives, limitations, multiplicity of analyses, results from similar studies, and other relevant evidence	7
Generalisability	21	Discuss the generalisability (external validity) of the study results	8
**Other information**			
Funding	22	Give the source of funding and the role of the funders for the present study and, if applicable, for the original study on which the present article is based	10

*Give information separately for cases and controls in case-control studies and, if applicable, for exposed and unexposed groups in cohort and cross-sectional studies.

Note: An Explanation and Elaboration article discusses each checklist item and gives methodological background and published examples of transparent reporting. The STROBE checklist is best used in conjunction with this article (freely available on the Web sites of PLoS Medicine at http://www.plosmedicine.org/, Annals of Internal Medicine at http://www.annals.org/, and Epidemiology at http://www.epidem.com/). Information on the STROBE Initiative is available at www.strobe-statement.org.

For peer review only

BMJ Open

COVID-19-related social support service closures and mental well-being in older adults and those affected by dementia: A UK longitudinal survey

Journal:	*BMJ Open*
Manuscript ID	bmjopen-2020-045889.R2
Article Type:	Original research
Date Submitted by the Author:	03-Jan-2021
Complete List of Authors:	Giebel, Clarissa; University of Liverpool, Institute of Psychology, Health and Society<br>Pulford, Daniel; Lancashire Care NHS Foundation Trust<br>Cooper, Claudia; University College London<br>Lord, Kathryn; University of Bradford<br>Shenton, Justine; Sefton Older People's Forum<br>Cannon, Jacqui; Lewy Body Society<br>Shaw, Lisa; University of Liverpool, Institute of Psychology, Health and Society<br>Tetlow, Hilary; SURF Liverpool<br>Limbert, Stan; NIHR ARC NWC<br>Callaghan, Steve; EQE Health<br>Whittington, Rosie; Me2U Care<br>Rogers, Carol; National Museums Liverpool<br>Komuravelli, Aravind; North West Boroughs Healthcare NHS Foundation Trust<br>Rajagopal, Manoj; Lancashire Care NHS Foundation Trust<br>Eley, Ruth; Liverpool DAA<br>Downs, Murna; University of Bradford, Bradford Dementia Group<br>Reilly, Siobhan; Lancaster University, Division of Health Research<br>Ward, Kym; The Brain Charity<br>Gaughan, Anna; TIDE<br>Butchard, Sarah; University of Liverpool, Institute of Psychology, Health and Society<br>Beresford, Jules; University of Bradford<br>Watkins, Caroline; University of Central Lancashire, Faculty of Health and Wellbeing<br>Bennett, Kate; University of Liverpool, Institute of Psychology, Health and Society<br>Gabbay, Mark
**Primary Subject Heading**:	Public health
Secondary Subject Heading:	Mental health
Keywords:	Dementia < NEUROLOGY, COVID-19, MENTAL HEALTH, Old age psychiatry < PSYCHIATRY

SCHOLARONE™
Manuscripts

I, the Submitting Author has the right to grant and does grant on behalf of all authors of the Work (as defined in the below author licence), an exclusive licence and/or a non-exclusive licence for contributions from authors who are: i) UK Crown employees; ii) where BMJ has agreed a CC-BY licence shall apply, and/or iii) in accordance with the terms applicable for US Federal Government officers or employees acting as part of their official duties; on a worldwide, perpetual, irrevocable, royalty-free basis to BMJ Publishing Group Ltd ("BMJ") its licensees and where the relevant Journal is co-owned by BMJ to the co-owners of the Journal, to publish the Work in this journal and any other BMJ products and to exploit all rights, as set out in our licence.

The Submitting Author accepts and understands that any supply made under these terms is made by BMJ to the Submitting Author unless you are acting as an employee on behalf of your employer or a postgraduate student of an affiliated institution which is paying any applicable article publishing charge ("APC") for Open Access articles. Where the Submitting Author wishes to make the Work available on an Open Access basis (and intends to pay the relevant APC), the terms of reuse of such Open Access shall be governed by a Creative Commons licence – details of these licences and which Creative Commons licence will apply to this Work are set out in our licence referred to above.

Other than as permitted in any relevant BMJ Author's Self Archiving Policies, I confirm this Work has not been accepted for publication elsewhere, is not being considered for publication elsewhere and does not duplicate material already published. I confirm all authors consent to publication of this Work and authorise the granting of this licence.

For peer review only

COVID-19-related social support service closures and mental well-being in older adults and those affected by dementia: A UK longitudinal survey

Clarissa Giebel[1,2,*], Daniel Pulford[3], Claudia Cooper[4], Kathryn Lord[5], Justine Shenton[6], Jacqueline Cannon[7], Lisa Shaw[8], Hilary Tetlow[9], Stan Limbert[2], Steve Callaghan[10], Rosie Whittington[11], Carol Rogers[12], Aravind Komuravelli[13], Manoj Rajagopal[3], Ruth Eley[14], Murna Downs[5], Siobhan Reilly[5], Kym Ward[15], Anna Gaughan[16], Sarah Butchard[17], Jules Beresford[5], Caroline Watkins[2,18], Kate Bennett[19], Mark Gabbay[1,2]

1 Department of Primary Care & Mental Health, University of Liverpool, UK
2 NIHR ARC NWC, Liverpool, UK
3 Lancashire and South Cumbria NHS Trust, UK
4 Division of Psychiatry, UCL, UK
5 Centre for Applied Dementia Studies, University of Bradford, UK
6 Sefton Older People's Forum, Sefton, UK
7 Lewy Body Society, Wigan, UK
8 Department of Modern Languages and Cultures, University of Liverpool, UK
9 SURF Liverpool, UK
10 EQE Health, Liverpool, UK
11 Me2U Care, Liverpool, UK
12 National Museums Liverpool, UK
13 North West Boroughs NHS Trust, Warrington, UK
14 Liverpool Dementia Action Alliance, UK
15 The Brain Charity, Liverpool, UK
16 TIDE, Liverpool, UK
17 Mersey Care NHS Trust, Liverpool, UK
18 Department of Health Research, UCLAN, UK
19 School of Psychology, University of Liverpool, UK

**Correspondence should be addressed to:* Clarissa Giebel, Waterhouse Building, University of Liverpool, Liverpool, L69 3GL. Email: Clarissa.giebel@liverpool.ac.uk

For peer review only

For peer review only - http://bmjopen.bmj.com/site/about/guidelines.xhtml

Abstract

Background: The COVID-19 pandemic has had a major impact on delivery of social support services. This might be expected to particularly affect older adults and people living with dementia (PLWD), and to reduce their wellbeing.

Aims: To explore how social support service use by older adults, carers, and PLWD, and their mental wellbeing changed over the first three months since the pandemic outbreak.

Methods: Unpaid dementia carers, PLWD, and older adults took part in a longitudinal online or telephone survey collected in April-May 2020, and at two subsequent time points 6 and 12 weeks after baseline. Participants were asked about their social support service usage in a typical week prior to the pandemic (at baseline), and in the past week at each of the three time points. They also completed measures of levels of depression, anxiety and mental well-being.

Results: 377 participants had complete data at all three time points. Social support service usage dropped shortly after lockdown measures were imposed at timepoint 1 (T1), to then increase again by T3. The access to paid care was least affected by COVID-19. Cases of anxiety dropped significantly across the study period, whilst cases of depression rose. Well-being increased significantly for older adults and PLWD from T1 to T3.

Conclusions: Access to social support services has been significantly affected by the pandemic, which is starting to recover slowly. With mental well-being differently affected across groups, support needs to be put in place to maintain better well-being across those vulnerable groups during the ongoing pandemic.

For peer review only

Strengths and limitations of this study

- Data on social support service usage and mental health were collected at three time points in the early stages of the pandemic
- 63% of participants completed all three survey time points
- The survey was co-produced with people affected by dementia
- The study was mostly completed by people from a White ethnic background, and lacks ethnic minority representation despite actively approaching community groups
- Some people with dementia completed the survey, who may have had difficulties in recalling the services they received pre-pandemic

For peer review only

For peer review only - http://bmjopen.bmj.com/site/about/guidelines.xhtml

Introduction

In the UK, 11.9 million people are aged 65 and over [1], with over 850,000 living with dementia [2]. Social support services, including day care centres, support groups, paid home carers, and community activities, such as singing or arts groups, are important for maintaining a good quality of life for older people and people living with dementia (PLWD) [3-4]. In view of an ageing population and increasing numbers of PLWD, easily accessible services are crucial to support people socially, as well as with their care needs.

The COVID-19 pandemic has affected these social support services significantly. Social isolation as a result of social distancing, lockdowns, and shielding is a huge concern for older people across the globe since the beginning of the pandemic [5-6], with similar issues highlighted early for PLWD [7]. However, there is still a dearth of evidence on the mental well-being and access to care for those in need.

Specifically, in the UK, a nationwide three-month lockdown was imposed on the 23rd of March. Older people were over-represented in the group who were clinically extremely vulnerable, that is at greatest risk of severe illness from COVID-19, who were asked to shield by the government until early August. All adults aged 70+ were classified as being at least moderate risk of severe illness from COVID-19 [8]. During the most restrictive, earlier period of lockdown, people were advised to only go outside once a day for essential food shopping, pharmacy visits, or to exercise. Non-essential shops were closed, and only started reopening in July. With additional social distancing for the general population, and use of personal protective equipment (PPE) for the health and social care taskforce in place, these measures significantly impact the social support services that PLWD, carers, and older adults could receive. Recent qualitative evidence has highlighted how PLWD and unpaid carers have faced a sudden crisis in terms of accessing social support services since the pandemic [9], and have faced difficult decisions whether to continue or discontinue paid carers entering the home of the PLWD, for fear of potential virus transmission [10]. Whilst these qualitative accounts provide rich information on the experiences of having accessed (or failed to access) social support services during the pandemic, there appears to be no empirical evidence to date quantifying those experiences and linking these with mental well-being.

The aim of this exploratory study was to explore the impacts of COVID-19 on social support service closures and longitudinal changes in the mental illness and well-being of older adults, PLWD, and unpaid carers. Considering the new emergence of this field and thus lack of previous evidence, we hypothesised that would be associated with reduced social support service provision, which in turn was hypothesised to be associated with poorer mental health.

Methods

Participants and recruitment

We recruited UK residents who were aged 18+. PLWD were eligible to take part if they had a diagnosis of dementia. Unpaid carers were eligible to take part if they were (current carers) or had been caring for a relative or friend with dementia (former carers). Older adults were eligible to take part if they were aged 65 years or older.

Participants were recruited via different social support services third sector organisations, such as peer support group organisations, carer networks, cultural dementia training programme organisations, and national dementia subtype specific organisations, and by contacting people on their email circulation lists, via newsletters and social media accounts. We also directly contacted people who were accessing regular services, such as support groups or older people fora, via telephone. This ensured that people without internet access were able to participate in this research. We also utilised *Join Dementia Research*, a UK-wide

For peer review only - http://bmjopen.bmj.com/site/about/guidelines.xhtml

national online register of PLWD, carers, older adults, and health volunteers who are interested in taking part in dementia and ageing research.

Ethical approval was obtained from the University of Liverpool prior to study begin [Ref: 7626].

Data collection

The study was completed at three time points (T1, T2, T3), 5 and 6 weeks apart, respectively. Participants could complete the survey either online or over the phone with a research team member who entered their details into the online survey on their behalf. Participants from T1 (baseline) were followed-up with the same mental well-being questionnaires at T2 and T3 and were followed-up either by telephone or email, depending on how they completed T1 survey. T1 ran from 17th April to 15th May (+/- 3 days). T2 ran from 29th May to 26th June (+/- 3 days). T3 ran from 10th July to 7th August (+/- 3 days).

Variables and tools

At T1, participants were asked about their background characteristics (including age, gender, ethnicity, postcode, living situation, type of dementia (if applicable), and employment). Postcode data were collected to generate an Index of Multiple Deprivation (IMD) quintile. IMD provides a measure of neighbourhood deprivation, taking into account income, education, crime, and health, amongst others. Quintile 1 indicates least deprived neighbourhoods, with quintile 5 indicating the most deprived neighbourhoods.

Service usage was measured by asking about pre-pandemic and current receipt of different social support services (including paid carers, support groups, befrienders, day care centres, respite, meal deliveries, transport, social activities, clinical mental health support, and clinical physical support) and equipment, such as hand rails or shower seats, as well as the weekly total hours of social support services. Pre-pandemic service usage was defined as use of social support services in a typical week before the pandemic.

Participants were also asked to complete the Personalised Health Questionnaire 9 (PHQ-9) [11] for levels of depressive symptoms, the Generalised Anxiety Disorder 7 (GAD-7) [12] for levels of anxiety symptoms, and the Short Warwick-Edinburgh Mental Well-Being Scale [13] (SWEMWBS) for quality of life. Higher scores on the PHQ-9, GAD-7, and the SWEMWBS indicated higher levels of depressive symptomatology, anxiety symptomatology, and quality of life, respectively. We categorised participants who scored of 10 or more on the PHQ-9 as 'depressed' and on the GAD-7 as 'anxious', as based on previous extensive research, indicating that these cut offs indicate general anxiety disorder and depression, respectively [14]. At T2 and T3, participants were asked again about their current levels of social support service receipt, weekly hours of support, equipment, as well as the PHQ-9, GAD-7, and SWEMWBS.

Data analysis

Data were analysed using SPSS 25, and the significance level was set at $p<0.05$. Participant demographic characteristics and social support service usage and mental well-being variables were analysed using frequency analysis. Chi-square tests were used to assess variations in the proportions of participants who were categorised as 'depressed' or 'anxious'. Repeated measures ANOVAs with Greenhouse Geisser posthoc correction were used to analyse differences between T1, T2, and T3 in GAD-7 total, PHQ-9 total, and SWEMWBS total scores. For this analysis, only participants with complete GAD-7, PHQ-9, and SWEMWBS were included (n=377). Paired samples t-tests were employed to compare the means of GAD-7,

For peer review only - http://bmjopen.bmj.com/site/about/guidelines.xhtml

PHQ-9, and SWEMWBS at T1 between those who completed all three time points (n=377) and those who dropped out after T1 or T2 (n=192). Bivariate correlation analysis was employed to assess whether changes in weekly social support service usage from pre-pandemic to T1 were associated with changes in GAD-7, PHQ-9, and SWEMWBS between T1 and T3.

Public involvement

Unpaid carers and a PLWD were involved as equal team members in all aspects of the study – from conceptualisation and design through to analysis and dissemination.

Results

Survey completion

Figure 1 outlines the participant flow and completion rates in further detail. 569 participants completed the survey at T1 (61 PLWD; 219 current carers; 66 former carers; 223 older adults). 420 participants completed the survey at T2 (38 PLWD; 168 current carers; 45 former carers; 169 older adults). 377 participants completed all three waves of the survey (37 PLWD; 149 current carers; 39 former carers; 152 older adults).

Participant characteristics

Table 1 shows the demographic characteristics of those who completed T1 and those that completed all three survey time points, by subgroup. For those who completed all three time points, carers and older adults were mostly female (59-82%), whilst the majority of PLWD were male (62%). The majority of participants were from a White ethnic background (95-99%) and lived with someone else (61-88%), with current carers having the highest proportion of living with someone else. The majority of participants across all four groups lived in less deprived neighbourhoods (Quintiles 1 and 2) (52-61%). Thirty-seven PLWD took part in all three time points; the most common diagnostic subtype was Alzheimer's disease.

[Figure 1 and Table 1]

Social support service and activities usage

Participants had accessed a range of social support services pre-pandemic, including day care centres, support groups, meal deliveries, respite, and paid carers. Figure 2 (A) shows the proportion of participants of the total sample (n=377) who reported accessing paid carers, support groups, day care, befrienders, and social activities prior to the pandemic, and at T1, T2, and T3. These were the most commonly used types of social support services prior to the pandemic. Social support services usage had dropped since the pandemic outbreak. Pre-pandemic, 27% of participants accessed social activities in the community, which dropped to 6% at T1, T2, and T3. Paid care saw the smallest change – with 17% having accessed paid carers pre-pandemic, dropping to 12% at T1 and increasing slightly again to 15% at T3. Day care saw the largest drop, with only 1-2 % receiving day care since the outbreak, compared to 15% previously.

Figure 2 (B) shows the proportion of participants by group who have received any form of social support services pre-pandemic and at all three survey time points. Pre-pandemic, 90% of current carers had received social support of any form, with between 45% and 50% of former carers and older adults having received some support. This decreased at T1 for all groups to between 20% (older adults) to 55% (current carers) receiving some type of support. Through T2 and T3, an upward trend emerged with more participants gaining access to some services again, with levels for PLWD and former carers being higher at T3 than at pre-pandemic levels.

[Figure 2]

For peer review only - http://bmjopen.bmj.com/site/about/guidelines.xhtml

Mental well-being

Figure 3 shows the proportion of participants across the total sample (n=377) who were categorised and identified as anxious and depressed, based on scoring above the GAD-7 and PHQ-9 cut off, across all three time points. For anxiety, we noted a downward trend in number of cases from T1 (16.5%) to T3 (14.1%). The proportion of participants with anxiety was significantly lower at T2 (x^2=186.399, p<0.001) and T3 (x^2=136.562, p<0.001) compared to T1. For depression, we noted an upward trend in cases, as indicated based on their cut off on the PHQ-9, from T1 (14.4%) to T3 (17.5%). The proportion of participants with depression was significantly higher at T2 (x^2= 176.248, p<0.001) and T3 (x^2=158.031, p<0.001) compared to T1.

Figure 4 shows the median of the GAD-7, PHQ-9, and SWEMWBS total scores by group over time for those who completed all three survey time points. Based on the median scores, levels of anxiety and depression appear to decrease from T1 to T3, whilst quality of life increases from T1 to T3.

Paired samples t-test showed that there were no significant differences in means of GAD-7 (p=.468), PHQ-9 (p=.183), and SWEMWBS (p=.332) at T1 between those who completed all three survey time points and those who dropped out after T1 or T2.

For anxiety, repeated-measures ANOVAs with Greenhouse-Geisser posthoc correction showed that GAD-7 total scores did not vary significantly from T1 to T3 for PLWD [F(1.856, 64.962)= 1.429, p= 0.247]or among current carers [F(1.898, 277.063)= 1.938, p=0.148], former carers[F(1.801, 68.419)=.139, p=.139], or older adults [F(1.924, 286.727)= 2.688, p=.0072], based on those participants who completed in all three survey time points.

For depression, repeated-measures ANOVAs showed that PHQ-9 total scores did not significantly vary from T1 to T3 for PLWD [F(1.896, 66.370)= 1.461, p=0.240], current carers [F(1.900, 277.453)= .639, p=0.521], former carers [F(1.677, 68.419)= .024, p=0.960], or older adults [F(1.889, 281.414)= .857, p=0.420].

For well-being, repeated-measures ANOVAs showed that SWEMWBS total scores significantly increased from T1 to T3 for PLWD [F(1.726, 60.423)= 5.412, p<0.05 [Mean(SD) T1-T3= 22.1(6.4); 24.3(5.6); 24.4(5.6)]] and older adults [F(1.804, 268.807)= 3.632, p<0.05 [Mean(SD) T1-T3= 27.5(5.1); 28.0(5.1); 28.3(4.9)]]. However there were no significant changes among current [F(1.982,289.325)=2.185, p=0.115] or former carers [F(1.728, 63.725)= .268, p=0.733].

[Figure 3 and 4]

Social support and mental health

Bivariate correlation analyses showed no significant associations between variations in social support service hours between pre-pandemic and at T1 and changes in SWEMWBS (p=.332), GAD-7 (p=.310), and PHQ-9 (p=.351) between T1 and T3 for those who completed all three survey time points.

Discussion

This is one of the first studies to show that social support service usage in dementia and ageing reduced significantly compared to pre-pandemic levels, whilst slowly rising in the months post-nationwide lockdown. In addition, we also show that cases of anxiety reduced whilst cases of depression increased in the months since lockdown, with quality of life significantly increasing for PLWD and older adults only.

Social support service usage for PLWD, unpaid carers, and older adults has seen a significant decrease since the COVID-19 pandemic, leaving many people suddenly without vital support – ranging from day care centres to respite to support groups. In the months

For peer review only - http://bmjopen.bmj.com/site/about/guidelines.xhtml

following the nationwide lockdown, usage has gradually increased again but varied among providers and type of support. With public health restrictions still remaining in place however during that period, including social distancing, shielding and thus inability to meet members of different households, such support is most likely to be implemented via digital technologies. Considering that in our sample 94% of participants completed the survey online rather than the telephone option, nearly all participants had access to the internet. However, many older adults and PLWD are less likely to be digitally literate [15], making it difficult for all people to access services equally. This has already been an issue pre-COVID-19 [16], suggesting that the pandemic has further exacerbated potential inequalities in access and thus further isolated people who would benefit from social support the most.

One type of support which has been affected the least by the pandemic has been paid home care. Receiving paid home care enables PLWD and older adults to stay at home independently for longer – as people wish to avoid entering a care home and stay in their familiar environment [17]. Whilst there was a reduction in paid home care usage compared to pre-pandemic levels, overall paid home carers were utilised the most. A qualitative exploration into decision-making for whether or not to continue paid home care during the pandemic has shown that many unpaid carers were afraid of having paid carers enter the home (often with inadequate PPE) for risk of potential virus transmission [10]. Other unpaid carers however felt unable to cope without the support, or indeed accepted the potential risks, and continued paid home care. There is also a notable difference between social care provision (which is paid home care) and third sector care provision (which involves support groups and social activities for example). The third sector relies on volunteers providing services, and has also suffered during the pandemic, whereas the social care sector is financially supported by the government. Therefore, the ability to receive home care might not have been affected to the same extent as accessing support groups for instance. Another potential reason for variations in usage between activity types is that home care involves someone from the outside entering someone's home. In contrast, day care centres, respite care, and social activities involve older adults and PLWD going outside to larger social gatherings. Due to public health restrictions, these have been temporarily rendered largely, if not completely, impossible to take place in their original face-to-face formats. As numbers of infections rise again, these restrictions are being strengthened and re-imposed with large fines possible for those transgressing them.

Levels of anxiety, depression, and well-being changed over the course of the study period. Over 12 weeks, cases of anxiety across the total sample dropped, whilst cases of depression increased significantly. However, when exploring levels of anxiety and depression within groups, no significant changes were noted, which is likely to have been due to small and varied sample sizes for each subgroup. Similarly, no significant variations in levels of anxiety, depression, and mental well-being were found between those who completed all three survey time points, and those who had dropped out after T1 or T2. It is possible that participants felt more connected over time, particularly considering again that the majority of participants completed the survey online and thus were able to participate in remote services, where these existed. Recent evidence from Spain showed how older adults were less likely to suffer from psychological distress as a result of the pandemic than people aged below 60 [18]. Nevertheless, overall the pandemic is having a heightened impact on the mental health of the general population [19-20]. Engaging in social activities can be one avenue to help maintain good mental health [21]. Considering that reductions in social engagement both before and after a dementia diagnosis are common [22], enabling continued engagement throughout the pandemic is important to support PLWD, carers, and older adults adequately. This is corroborated by evidence from the baseline survey showing that reductions in social support usage were linked to mental well-being [23]. It is possible that for this study, merging groups of older adults, PLWD, and carers resulted in no significant associations, as each group was

For peer review only - http://bmjopen.bmj.com/site/about/guidelines.xhtml

differently affected, as indicated by looking at changes of mental well-being for each group across the 12 weeks.

There were some limitations to our exploratory study. Whilst benefitting from a large sample size and good retention rate over a relatively short time period of 12 weeks, there was some missing data and not everyone completed all three survey time points. However, this is standard in longitudinal survey-based research, and we still generated a large sample size across all three time points. By comparing those who completed all three survey time points and those who dropped out after T1 or T2, we established that there no significant differences in their mental health scores. Concerning the participant population, it is to be noted that the majority of participants had internet access and were thus also able to join in remote social support. Although we actively approached older adults, PLWD, and carers via phone through recruiting organisations, only some people took part over the phone. It is likely however that those people without internet access have been even more isolated through the pandemic, with potentially severe mental health needs, which we have only captured a snapshot of. This also links to the fact that there are likely to be longer-term effects on mental well-being, with our survey only providing a snap shot of the first few months since the start of the pandemic. Equally, our survey did not include pre-pandemic levels of mental well-being (anxiety, depression, and quality of life), which would have provided additional insight into changes in mental well-being. However, due to the unforeseen circumstance of the pandemic, it was not feasible to collect these data. We only enquired about weekly hours of total social support usage, and not for each specific type of activity. Some participants might have accessed, for example, paid home care, but only for two hours as opposed to others who might have received 40 hours a week. We are thus unable to state in detail how the pandemic has affected the level of each different type of support, but instead we provide a more general overview of activities and general service usage variations since the nationwide lockdown, which to existing knowledge has not been captured elsewhere.

Conclusions

The pandemic is having a sudden and severe long-term impact on social support service usage for older adults and people affected by dementia, which sees somewhat of a limited increase in usage over the first few months since nationwide lockdown. Whilst it appears that some services have started providing remote support, not everyone will be able to access these, leaving many people without much needed support. Future research needs to assess how older adults and people affected by dementia are accessing social support services in the time of COVID-19, with clearer support for people to access any format of services – either face-to-face or remotely. Considering that the pandemic is going to continue for the foreseeable future, the mental health of older adults and those affected by dementia needs to be closely monitored, particularly when more stringent public health measures are put in place again.

For peer review only - http://bmjopen.bmj.com/site/about/guidelines.xhtml

Conflicts of interest
None.

Acknowledgements
This study would not have been possible without our participants, thank you very much. We also wish to thank Join Dementia Research for helping us recruit to our study.

Funding
This research is supported by a grant awarded to the authors by the University of Liverpool COVID-19 Strategic Research Fund in 2020. This is also independent research funded by the National Institute for Health Research Applied Research Collaboration North West Coast (ARC NWC). The views expressed in this publication are those of the author(s) and not necessarily those of the National Institute for Health Research or the Department of Health and Social Care. The University of Bradford QR Research Fund also supported part of this study. There are no grant numbers.

Data availability statement
Data may be obtained from a third party and are not publicly available.

Author contributions
CG managed data collection, conducted analysis, and wrote the manuscript. JC, JS, and DP collected data over the phone. DP managed the data. All authors (CG, DP, CC, KL, JS, JC, LS, HT, SL, SC, RW, CR, AK, MR, RE, MD, SR, KW, AG, SB, JB, CW, KB, MG) contributed to designing the survey, interpreting the findings, and reading drafts of the manuscript. All authors approved the final manuscript. Emma Riley helped with recruitment by emailing eligible participants via the House of Memories networks and we wish to thank her for her support with this.

For peer review only

Figure Captions

Figure 1. Flow of participation in longitudinal survey

NOTE. The top boxes indicate how many people completed each survey time point. After having removed (1) duplicates (people who completed the survey twice), (2) missing cases (where participants had not completed the PHQ-9, GAD-7, and the SWEMWBS or had missing ID codes at T2 and T3), and (3) losses to follow-up (those that had either completed T1 or T1 and T2 only), and (4) incomplete data at T1 yet data at T2 or T3, 377 cases remained in total. Grey boxes indicate the breakdown by subgroup.

[1] Follow-up completion by subgroup by percentage at T2 and T3 compared to T1: PLWD 69%(T2), 67%(T3); Current Carers 86%(T2), 75%(T3); Former carers 64%(T2), 60%(T3); Older adults 79%(T2), 69%(T3).

Figure 2. Social support service usage pre-pandemic and at 3 survey time points

Note. (A) Service usage for the total sample (N=377) in proportion of participants at four different time points for some of the most frequently used support services. (B) Proportion of participants within each group at four different timepoints (pre-pandemic, T1, T2, T3) having received any form of social support.

Figure 3. Proportion of the total sample who scored above the cut offs for anxiety and depression at three time points

NOTE. T = Time point

The graph shows the proportion of participants from the total sample who completed all three surveys (n=377) and scored above the cut off on the GAD-7 and PHQ-9 for anxiety and depression, respectively.

Figure 4. Variations in anxiety, depression, and quality of life total scores at 3 time points

NOTE. Figures show the median total score at each time point (T1, T2, T3) for each subgroup – for anxiety (GAD-7), depression (PHQ-9), and quality of life (SWEMWBS).

For peer review only

References

[1] Office for National Statistics (ONS). *Living longer: is age 70 the new age 65?* London, England 2019: ONS.

[2] Alzheimer's Society. *Dementia UK: An Update*. London, England: Alzheimer's Society 2014.

[3] Skingley A, Martin A, Clift S. The contribution of community singing groups to the well-being of older people: participant perspectives from the United Kingdom. Journal of Applied Gerontology 2016;35(12):1302-1324.

[4] Tretteteig S, Vatne S, Mork Rokstad AM. The influence of day care centres designed for people with dementia on family caregivers – a qualitative study. BMC Geriatrics 2017;17(5).

[5] Armitage R, Nellums LB. COVID-19 and the consequences of isolating elderly. LANCET Public Health 2020; 5(5):doi: 10.1016/S2468-2667(20)30061-X

[6] Berg-Weger M, Morley JE. Loneliness and social isolation in older adults during the COVID-19 pandemic: implications for gerontological social work. Journal of Nutrition, Health & Ageing 2020;24(5):456-458.

[7] Brown EE, Kumr S, Rajji TK, et al. Anticipating and mitigating the impact of the COVID-19 pandemic on Alzheimer's disease and related dementias. American Journal of Geriatric Psychiatry 2020;28(7):712-721.

[8] Public Health England (PHE). *Guidance on shielding and protecting people who are clinically extremely vulnerable from COVID-19.* London, England 2020: PHE. Last accessed 22nd September 2020: https://www.gov.uk/government/publications/guidance-on-shielding-and-protecting-extremely-vulnerable-persons-from-covid-19/guidance-on-shielding-and-protecting-extremely-vulnerable-persons-from-covid-19#definition

[9] Giebel C, Cannon J, Hanna K, et al. Impact of Covid-19 related social support service closures on people with dementia and unpaid carers: A qualitative study. Aging & Mental Health 2020a; doi: 10.1080/13607863.2020.1822292

[10] Giebel C, Hanna K, Cannon J, et al. Decision-making for receiving paid home care for dementia in the time of COVID-19: A qualitative study. BMC Geriatrics 2020b; doi: 10.1186/s12877-020-01719-0

[11] Kroenke K, Spitzer RL, Williams JB. The PHQ-9: validity of a brief depression severity measure. Journal of General Internal Medicine 2001;16(9):606-613.

[12] Spitzer RL, Kroenke K, Williams JB, Loewe B. A brief measure for assessing generalized anxiety disorder: the GAD-7. Archives of Internal Medicine 2006;166(10):1092-1097.

[13] Haver A, Akerjordet K, Caputi P, et al. Measuring mental well-being: A validation of the Short Warwick-Edinburgh Mental Well-Being Scale in Norwegian and Swedish. Scandinavian Journal of Public Health 2015;43(7):721-727.

[14] Smith, B.W., Epstein, E.E., Oritz, J.A., Christopher, P.K., & Tooley, E.M. (2013). The Foundations of Resilience: What are the critical resources for bouncing back from stress? In Prince-Embury, S. & Saklofske, D.H. (Eds.), *Resilience in children, adolescents, and adults: Translating research into practice*, The Springer series on human exceptionality (pp. 167-187). New York, NY: Springer.

For peer review only - http://bmjopen.bmj.com/site/about/guidelines.xhtml

For peer review only

[15] Hill R, Betts LR, Gardner SE. Older adults' experiences and perceptions of digital technology: (Dis)empowerment, wellbeing, and inclusion. Computers in Human Behavior 2015;48: 415-423.

[16] Stephan A, Bieber A, Hopper L, et al. Barriers and facilitators to the access to and use of formal dementia care: findings of a focus group study with people with dementia, informal carers and health and social care professionals in eight European countries. BMC Health Services Research 2018; 131: doi: 10.1186/s12877-018-0816-1

[17] Rapaport P, Burton A, Leverton M, et al. "I just keep thinking that I don't want to rely on people." A qualitative study of how people living with dementia achieve and maintain independence at home: stakeholder perspectives BMC Geriatrics 2020; doi: 10.1186/s12877-019-1406-6

[18] Garcia-Portilla P, de la Fuente Tomas L, Bobes-Bascaran T, et al. Are older adults also at higher psychological risk from COVID-19? Aging & Mental Health 2020; doi: 10.1080/13607863.2020.1805723

[19] Fancourt D, Steptoe A, Bu F. Trajectories of depression and anxiety during enforced isolation due to COVID-10: longitudinal analyses of 59,318 adults in the UK with and without diagnosed mental illness. medRxiv preprint 2020; doi: 10.1101/2020.06.03.20120923

[20] Iob E, Steptoe A, Fancourt D. Abuse, self-harm and suicidal ideation in the UK during the COVID-19 pandemic. British Journal of Psychiatry 2020; doi: 10.1192/bjp.2020.130

[21] Holtfreter K, Reisig MD, Turanovic JJ. Depression and infrequent participation in social activities among older adults: the moderating role of high-quality familial ties. Aging & Mental Health 2017;21(4): 10.1080/13607863.2015.1099036

[22] Hackett RA, Steptoe A, Cadar D, Fancourt D. Social engagement before and after dementia diagnosis in the English Longitudinal Study of Ageing. PLOS One 2019; doi: 10.1371/journal.pone.0220195

[23] Giebel C, Lord K, Cooper C, et al. A UK survey of COVID-19 related social support service closures and the effects on older people, people with dementia, and carers. International Journal of Geriatric Psychiatry 2020c; doi: 10.1002/gps.5434

For peer review only - http://bmjopen.bmj.com/site/about/guidelines.xhtml

Table 1. Participant characteristics of those completing T1 survey and those completing all three survey time points

	T1 (n=569)				T1, 2 and 3 (n=377)			
	**PLWD (n=61)**	**Current carers (n=219)**	**Former carers (n=66)**	**Older adults (n=223)**	**PLWD (n=37)**	**Current carers (n=149)**	**Former carers (n=39)**	**Older adults (n=152)**
**N(%)**								
**Gender**								
**Female**	27 (44.3)	168 (77.1)	55 (83.3)	137 (61.7)	14 (37.8)	118 (79.7)	32 (82.1)	90 (59.2)
**Male**	34 (55.7)	50 (22.9)	11 (16.7)	85 (38.3)	23 (62.2)	30 (20.3)	7 (17.9)	62 (40.8)
**Ethnicity**								
**White**	58 (96.7)	211 (96.3)	65 (98.5)	216 (98.2)	35 (94.6)	143 (96.0)	38 (97.4)	148 (98.7)
**Other**	2 (3.4)	8 (3.7)	1 (1.5)	4 (1.9)	2 (5.4)	6 (4.0)	1 (2.6)	2 (1.3)
**Living situation**								
**Alone**	13 (21.3)	33 (15.1)	17 (26.2)	79 (35.6)	8 (21.6)	18 (12.2)	11 (28.9)	59 (39.1)
**With someone**	48 (78.7)	185 (84.9)	48 (73.8)	143 (64.4)	29 (78.4)	130 (87.8)	27 (71.1)	92 (60.9)
**IMD Quintile**								
**1**	12 (23.1)	54 (32.1)	10 (19.2)	61 (33.5)	5 (16.1)	35 (31.0)	6 (19.4)	39 (31.7)
**2**	16 (30.8)	50 (29.8)	20 (38.5)	44 (24.2)	11 (35.5)	34 (30.1)	11 (35.5)	29 (23.6)
**3**	10 (19.2)	32 (19.0)	14 (26.9)	37 (20.3)	6 (19.4)	20 (17.7)	9 (29.0)	26 (21.1)
**4**	10 (19.2)	14 (8.3)	5 (9.6)	26 (14.3)	5 (16.1)	11 (9.7)	4 (12.9)	18 (14.6)
**5**	4 (7.7)	18 (10.7)	3 (5.8)	14 (7.7)	4 (12.9)	13 (11.5)	1 (3.2)	11 (8.9)
**Type of dementia**								
**Alzheimer's**	20 (32.8)	100 (46.5)	6 (23.1)		14 (37.8)	75 (50.7)	2 (20.0)	
**Mixed**	13 (21.3)	49 (22.8)	7 (26.9)		6 (16.2)	34 (23.0)	4 (40.0)	
**Vascular**	11 (18.0)	27 (12.6)	4 (15.4)		8 (21.6)	18 (12.2)	3 (30.0)	
**Other**	17 (27.9)	39 (18.1)	9 (34.5)		9 (24.3)	21 (14.1)	1 (10.0)	
**Mean (SD), [Range]**								
**Age**	70 (+/-10), [45-88]	61 (+/-13), [23-89]	64 (+/-14), [22-95]	72 (+/-6), [65-90]	72 (+/-10), [50-88]	62 (+/-13), [23-89]	65 (+/-13), [22-95]	73 (+/-6), [65-90]
**Years of education**	15 (+/-4), [4-25]	16 (+/-4), [6-28]	17 (+/-4), [10-29]	17 (+/-4), [7-25]	13 (+/-4), [4-20]	16 (+/-4), [6-28]	16 (+/-4), [10-29]	16 (+/-4), [7-24]

NOTE. 569 participants completed the survey at T1, with duplicates and missing cases removed. 377 participants had completed all three survey time points, with duplicates and missing cases removed.

PLWD – People living with dementia

For peer review only - http://bmjopen.bmj.com/site/about/guidelines.xhtml

For peer review only

For peer review only - http://bmjopen.bmj.com/site/about/guidelines.xhtml

Figure 1. Flow of participation in longitudinal survey

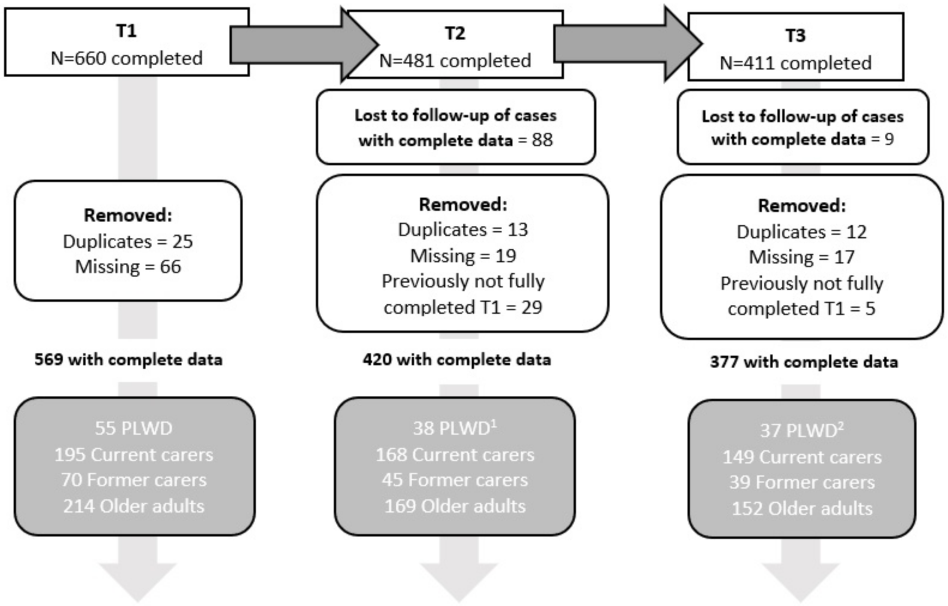

147x102mm (144 x 144 DPI)

Figure 2. Social support service usage pre-pandemic and at 3 survey time points

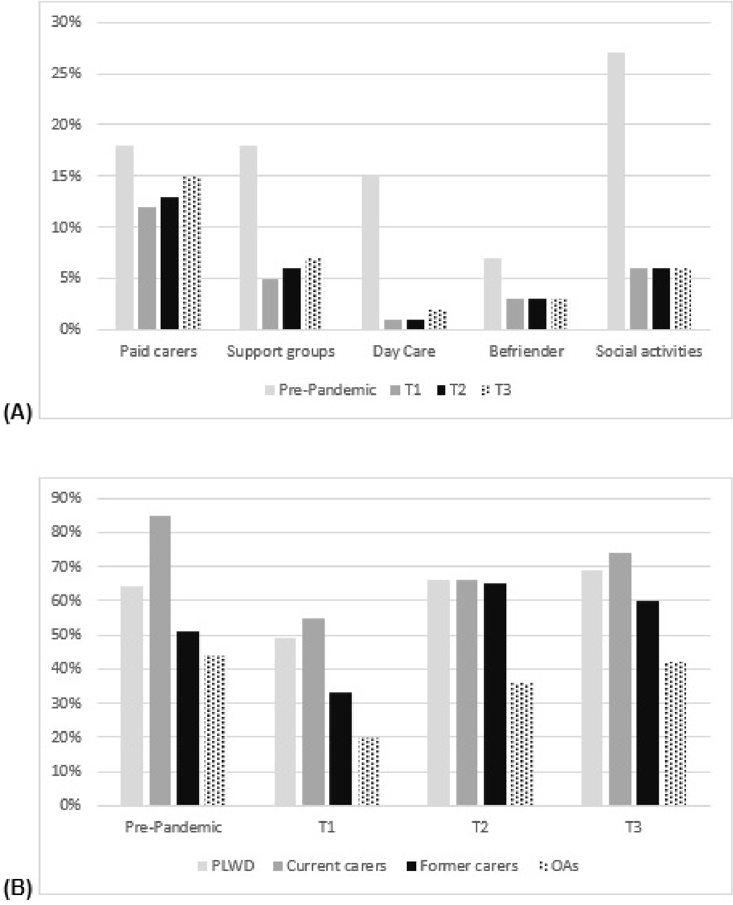

109x112mm (144 x 144 DPI)

Figure 3. Proportion of the total sample who scored above the cut offs for anxiety and depression at three time points

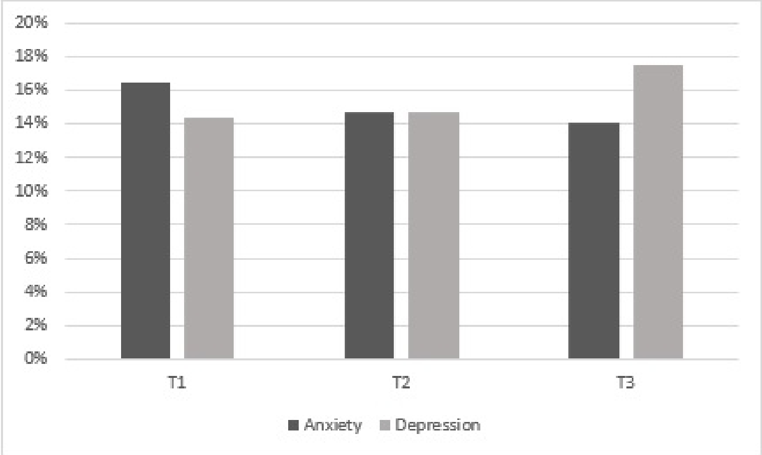

100x54mm (144 x 144 DPI)

Figure 4. Variations in anxiety, depression, and quality of life total scores at 3 time points

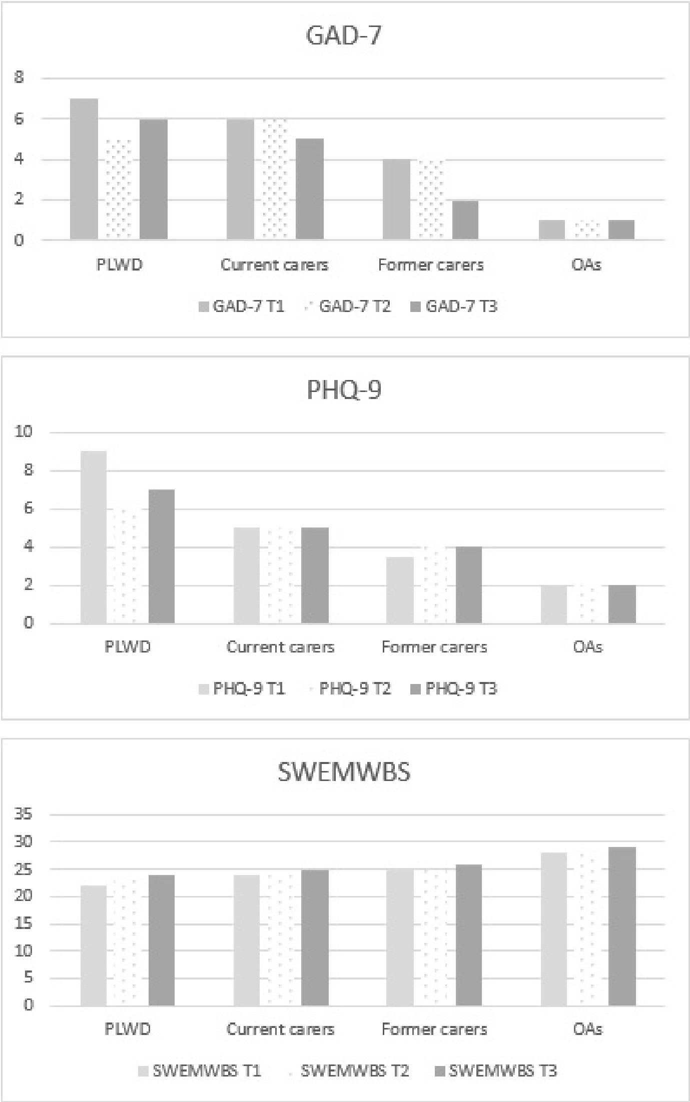

101x121mm (144 x 144 DPI)

STROBE Statement—checklist of items that should be included in reports of observational studies

	Item No	Page	Recommendation
**Title and abstract**	1	1 & 2	(*a*) Indicate the study's design with a commonly used term in the title or the abstract
		1 & 2	(*b*) Provide in the abstract an informative and balanced summary of what was done and what was found
		**Introduction**	
Background/rationale	2	4	Explain the scientific background and rationale for the investigation being reported
Objectives	3	4	State specific objectives, including any prespecified hypotheses
		**Methods**	
Study design	4	4-5	Present key elements of study design early in the paper
Setting	5	4-5	Describe the setting, locations, and relevant dates, including periods of recruitment, exposure, follow-up, and data collection
Participants	6	4-5	(*a*) *Cohort study*—Give the eligibility criteria, and the sources and methods of selection of participants. Describe methods of follow-up<br>*Case-control study*—Give the eligibility criteria, and the sources and methods of case ascertainment and control selection. Give the rationale for the choice of cases and controls<br>*Cross-sectional study*—Give the eligibility criteria, and the sources and methods of selection of participants
		4-5	(*b*) *Cohort study*—For matched studies, give matching criteria and number of exposed and unexposed<br>*Case-control study*—For matched studies, give matching criteria and the number of controls per case
Variables	7	5	Clearly define all outcomes, exposures, predictors, potential confounders, and effect modifiers. Give diagnostic criteria, if applicable
Data sources/ measurement	8*	5	For each variable of interest, give sources of data and details of methods of assessment (measurement). Describe comparability of assessment methods if there is more than one group
Bias	9	n/a	Describe any efforts to address potential sources of bias
Study size	10	6	Explain how the study size was arrived at
Quantitative variables	11	5	Explain how quantitative variables were handled in the analyses. If applicable, describe which groupings were chosen and why
Statistical methods	12	5	(*a*) Describe all statistical methods, including those used to control for confounding
		5	(*b*) Describe any methods used to examine subgroups and interactions
			(*c*) Explain how missing data were addressed
			(*d*) *Cohort study*—If applicable, explain how loss to follow-up was addressed<br>*Case-control study*—If applicable, explain how matching of cases and controls was addressed<br>*Cross-sectional study*—If applicable, describe analytical methods taking account of sampling strategy
			(*e*) Describe any sensitivity analyses

For peer review only

Continued on next page

For peer review only

For peer review only - http://bmjopen.bmj.com/site/about/guidelines.xhtml

**Results**			**Page**
Participants	13*	(a) Report numbers of individuals at each stage of study—eg numbers potentially eligible, examined for eligibility, confirmed eligible, included in the study, completing follow-up, and analysed	6
		(b) Give reasons for non-participation at each stage	
		(c) Consider use of a flow diagram	Figure 1
Descriptive data	14*	(a) Give characteristics of study participants (eg demographic, clinical, social) and information on exposures and potential confounders	6
		(b) Indicate number of participants with missing data for each variable of interest	6
		(c) *Cohort study*—Summarise follow-up time (eg, average and total amount)	
Outcome data	15*	*Cohort study*—Report numbers of outcome events or summary measures over time	*6*
		Case-control study—Report numbers in each exposure category, or summary measures of exposure	
		Cross-sectional study—Report numbers of outcome events or summary measures	
Main results	16	(*a*) Give unadjusted estimates and, if applicable, confounder-adjusted estimates and their precision (eg, 95% confidence interval). Make clear which confounders were adjusted for and why they were included	6-7
		(*b*) Report category boundaries when continuous variables were categorized	6-7
		(*c*) If relevant, consider translating estimates of relative risk into absolute risk for a meaningful time period	
Other analyses	17	Report other analyses done—eg analyses of subgroups and interactions, and sensitivity analyses	6-7
**Discussion**			
Key results	18	Summarise key results with reference to study objectives	7
Limitations	19	Discuss limitations of the study, taking into account sources of potential bias or imprecision. Discuss both direction and magnitude of any potential bias	8
Interpretation	20	Give a cautious overall interpretation of results considering objectives, limitations, multiplicity of analyses, results from similar studies, and other relevant evidence	7
Generalisability	21	Discuss the generalisability (external validity) of the study results	8
**Other information**			
Funding	22	Give the source of funding and the role of the funders for the present study and, if applicable, for the original study on which the present article is based	10

*Give information separately for cases and controls in case-control studies and, if applicable, for exposed and unexposed groups in cohort and cross-sectional studies.

Note: An Explanation and Elaboration article discusses each checklist item and gives methodological background and published examples of transparent reporting. The STROBE checklist is best used in conjunction with this article (freely available on the Web sites of PLoS Medicine at http://www.plosmedicine.org/, Annals of Internal Medicine at http://www.annals.org/, and Epidemiology at http://www.epidem.com/). Information on the STROBE Initiative is available at www.strobe-statement.org.

For peer review only

For peer review only - http://bmjopen.bmj.com/site/about/guidelines.xhtml
